# The Impact of Incomplete Taxon Sampling on Inference of Gene Flow by Bayesian and Summary Methods Using Genomic Sequence Data

**DOI:** 10.1093/sysbio/syag023

**Published:** 2026-03-03

**Authors:** Sirui Cheng, Thomas Flouri, Tianqi Zhu, Ziheng Yang

**Affiliations:** Department of Genetics, Evolution, and Environment, University College London, Gower Street, London WC1E 6BT, UK; State Key Laboratory of Mathematical Science, Academy of Mathematics and Systems Science, Chinese Academy of Sciences, Beijing 100190, China; University of Chinese Academy of Sciences, Beijing 100049, China; Department of Genetics, Evolution, and Environment, University College London, Gower Street, London WC1E 6BT, UK; State Key Laboratory of Mathematical Science, Academy of Mathematics and Systems Science, Chinese Academy of Sciences, Beijing 100190, China; Department of Genetics, Evolution, and Environment, University College London, Gower Street, London WC1E 6BT, UK

**Keywords:** Bayesian test, BPP, ghost introgression, introgression, migration, multispecies coalescent, Savage–Dickey, taxon sampling

## Abstract

Interspecific gene flow is commonly inferred using genomic data under the multispecies coalescent model. Incomplete taxon sampling can impact inference of gene flow in multiple ways. First unsampled ghost lineages that are sources of introgression may mislead inference of gene flow in analysis of genomic data from sampled species. Second incomplete taxon sampling causes merges of branches on the species phylogeny and complicates the definition and estimation of the rate or magnitude of gene flow, measured by the expected proportion of immigrants in the recipient population (i.e., the introgression probability). We use mathematical analysis and computer simulation to examine the impact of incomplete taxon sampling on inference of gene flow and estimation of its rate using genomic data. We introduce a Bayesian testing approach to select models of gene flow for a species triplet (such as ghost introgression, inflow, and outflow), using the Savage–Dickey density ratio to calculate Bayes factors. We show that the approach has excellent sensitivity and specificity, whereas heuristic methods based on data summaries typically cannot distinguish among those scenarios. We find that genomic data allow reliable estimation of the proportion of immigrants (rather than the number of immigrants), even when the assumed demographic model is incorrect due to incomplete taxon sampling. When population size differs among species, assuming the same size may lead to seriously biased estimates of the rate of gene flow. The *f*-branch approach is effective in reducing the number of gene-flow events suggested by triplet analyses but often fails to identify the correct model of gene flow and tends to underestimate the rate of gene flow. Our results highlight the need for improving summary methods to accommodate different population sizes and to infer gene flow between sister lineages.

Genomic sequences sampled from extant species provide a rich source of information concerning historical gene flow between species. Indeed gene flow has been detected using genomic data from a variety of species including animals as well as plants (Harrison and Larson 2014; Edelman and Mallet 2021). Gene flow has most often been inferred using simple summary methods that operate on species triplets (or quartets if an outgroup is included). They make use of either genome-wide site-pattern counts, as in the case of the *D*-statistic or ABBA-BABA test (Green et al. 2010) and HyDe (Blischak et al. 2018), or reconstructed gene tree topologies, as in SNaQ (Solis-Lemus and Ane 2016) and PhyloNet/MPL (Yu and Nakhleh 2015). An issue of incomplete taxon sampling arises as the analysis uses only three species/populations, with one sequence sampled per species, even if genomic data are available from many species and multiple samples per species. Another scenario arises when there exist ghost lineages, i.e., extinct or unsampled lineages that contributed genetic materials to modern sampled species or their ancestors. In this study we use the term incomplete taxon sampling to refer to both scenarios. Note that biases and errors resulting from a triplet analysis ignoring ghost gene flow are exactly the same whether the ghost lineages are (a) extant, sampled but unused; (b) extant but unsampled; or (c) extinct. Note also that unsampled lineages that are not donors of gene flow to sampled species (or their ancestors) do not impact on the analysis of data from the sampled species.

Incomplete taxon sampling may influence our inference of interspecific gene flow in multiple ways. First, the presence of ghost lineages may mislead our inference of gene flow in analysis of sampled species (Beerli 2004; Ottenburghs 2020). Consider the phylogeny for six species with gene flow of Fig. 1a. In analysis of the triplet *ACD*, species *E* and *F* are ghost lineages, which contribute genes to the sampled species *D*. Through simulations Tricou et al. (2022) found that introgression from an outgroup ghost lineage can cause the *D*-statistic to mis-identify the donor and the recipient populations involved in introgression. We note that the simulation of Tricou et al. (2022) failed to account for the coalescent process and did not follow standard procedures for frequentist simulation, as they sampled parameters such as species split times at random for each replicate dataset whereas correctly replicate datasets should be generated with parameter values fixed. Nevertheless the authors’ overall conclusion appears to be valid. Huang et al. (2022, Figs. 6 and 8) found that the absence of an intermediate ghost species does not have a great impact on bpp inference of gene flow, which detects ‘indirect’ gene flow via unsampled intermediate species as well as direct gene flow. Recently Pang and Zhang (2024) made the disturbing finding that commonly used triplet methods such as the *D*-statistic or ABBA-BABA test (Green et al. 2010), HyDe (Blischak et al. 2018), SNaQ (Solis-Lemus and Ane 2016), and PhyloNet/MPL (Yu and Nakhleh 2015) cannot distinguish among different scenarios of introgression on a triplet tree, such as introgression from an outgroup ghost species, and inflow or outflow between non-sister ingroup species (Figs. 2b–d). Those methods use two functions of the site-pattern frequencies (in the case of *D* and HyDe) or gene-tree proportions (in the case of SNaQ) to estimate two parameters in the triplet model (introgression probability *φ* and the internal branch length in coalescent units), exhausting all degrees of freedom in the data and leaving no power to distinguish among different models of gene flow.

**Figure 1.**
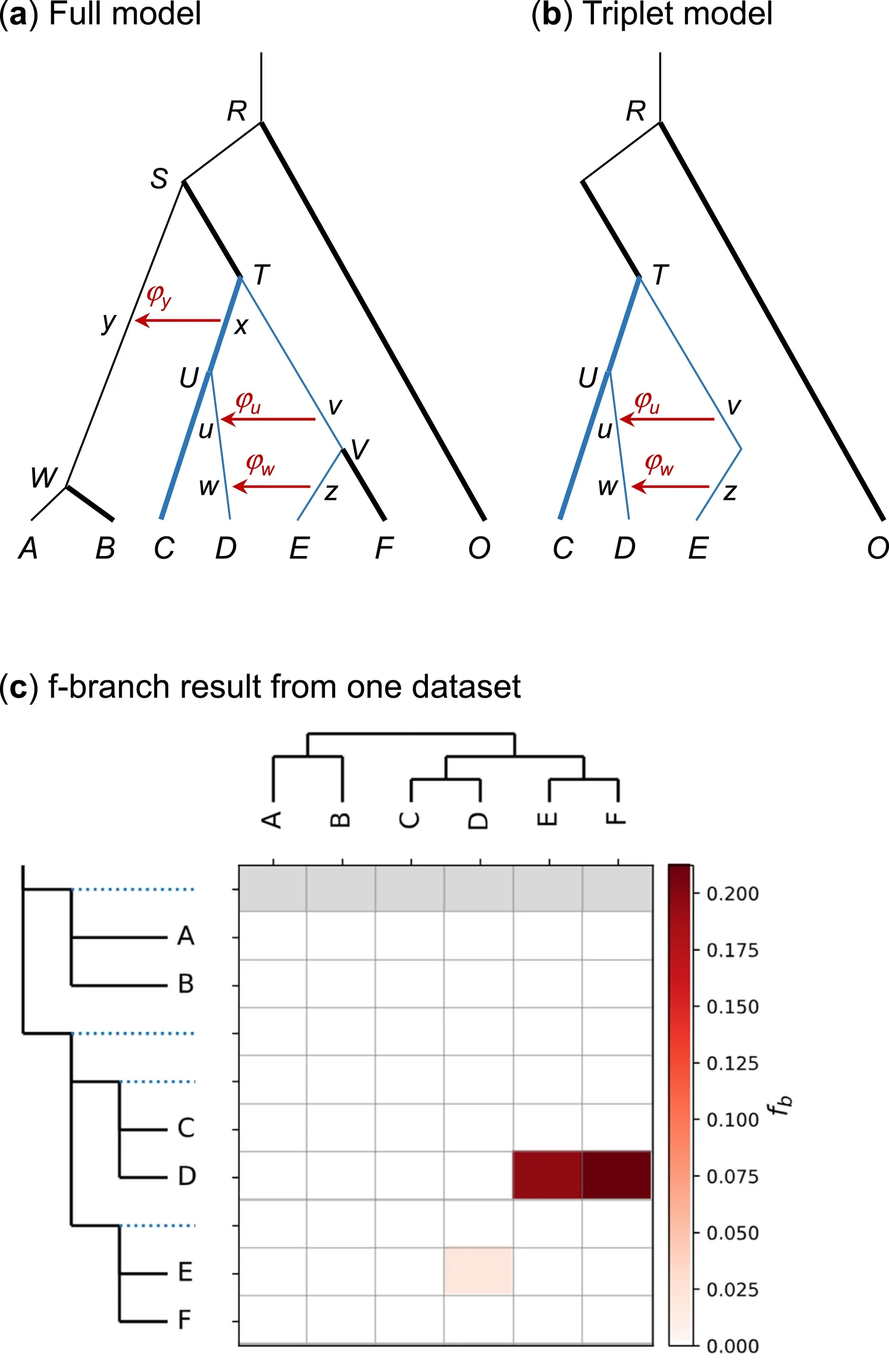
(a) Phylogeny for six species (*A*–*F*) plus an outgroup (*O*) with three introgression events used to simulate data. Population size was assumed to be either equal for all branches ($\theta _0=\theta _1 = 0.001$) or different for thin and thick branches ($\theta _0=0.001$ and $\theta _1=0.01$, respectively). (b) Triplet species tree for the *CDE* triplet (with outgroup *O*) after other species in the tree of panel **a** are pruned off. Branches *TV* and *VE* in the original tree are merged into one branch *TE* in the triplet tree. (c) Heatmap of introgression probabilities produced in the *f*-branch analysis of a replicate dataset simulated under the model of panel **a**, with *L=1000* loci (with *S = 4* sequences per locus and *n = 500* sites per sequence). Three introgression events were inferred: $f_D(F) = 0.212$, $f_D(E) = 0.194$, and $f_E(D) = 0.0194$.

Second, multiple branches on the phylogeny, which correspond to species with different population sizes, may be merged into one branch in the triplet tree and assigned one population size parameter. Biologically, one expects population size to fluctuate over time in response to geological and environmental changes such as mountain building, river reversals, ice ages, etc., but it is conventional to assign a constant population size for each species on the phylogeny under the multispecies coalescent model (e.g., Takahata et al. 1995; Rannala and Yang 2003). The rate of gene flow is typically measured by the introgression probability, $\varphi _{xy}$ — also denoted *γ* in HyDe (Blischak et al. 2018), SNaQ (Solis-Lemus and Ane 2016) and PhyloNet/MPL (Yu and Nakhleh 2015) and *f* in admixtools (Patterson et al. 2012) — and defined as the probability of immigrants in the recipient population *y* (from the donor population *x*) at the time of hybridization/introgression. Note that commonly used triplet methods for inferring introgression such as HyDe and SNaQ assume a constant population size across the phylogeny, and may be biased if population size changes over time or differs among lineages. The apparent changes in the definition of the recipient population or in its size due to exclusion of species on the phylogeny may affect our definition and estimation of the rate of gene flow. For example, is it the proportion or the number of immigrants that affect the genomic data and can thus be reliably estimated using genomic data? Because the introgression probability $\varphi _{xy}$ is also the probability that a sequence in the recipient population *y* is traced to the donor population *x* when one traces the genealogical history of the sample of sequences backwards in time, and because sequence data reflect gene genealogies, one may expect inference using sequence data to be dominated by the expected proportion of immigrants ($\varphi _{xy}$), rather than the expected number of immigrants (i.e., $N_y\varphi _{xy}$). This expectation is yet to be confirmed.

Third, branches in the phylogeny that are merged in the triplet tree may be involved in introgression in different directions at different time points, and those events are all merged into one introgression event with a single introgression probability, the expected value of which is poorly understood. For example, when we analyze the *CDE* triplet (Fig. 1b), the two introgression events, *v→ u* and *z→ w*, are merged into one event (from *E* to *D*). Do we expect the estimated introgression probability $\varphi _{E\rightarrow D}$ to be $\varphi _{vu} + \varphi _{zw}$? What if the introgressions are in opposite directions (*u→ v* and *z→ w*, say)?

Fourth, one gene flow event involving ancestral branches on the phylogeny may appear in many triplets, and it may be challenging to combine many triplet estimates into one introgression probability for the shared gene-flow event. For example, the *x→ y* introgression on the species tree of Fig. 1a may show up in eight triplets, $\binom{A}{B} \binom{C}{D} \binom{E}{F}$, generated by selecting one of *A* and *B*, one of *C* and *D*, and one of *E* and *F*, and the eight estimates may differ from the true rate, affected by different population sizes on the triplet trees and by random sampling errors.

Indeed the *D*-statistic has been observed to detect gene flow in overwhelmingly many triplets in analyses of empirical data (e.g., Malinsky et al. 2018). The challenge of interpreting such triplet results motivated the development of the *f*-branch approach, which assembles results from the triplet analyses (Malinsky et al. 2018, 2021). The method assumes a binary species tree and attempts to move gene-flow events onto ancestral branches which may explain the detection of gene flow in many triplets. Ideally one would like each triplet analysis to reflect the true model, and the assembled results from all triplets to recover the true model for the full phylogeny. Currently this ideal is not fulfilled, but we lack an understanding of the behavior of triplet methods when the three species are a subset of many species (as in the case of the *CDE* triplet of Fig. 1b when the true model is the one for six species of Fig. 1a). The inability of triplet methods to distinguish among competing models of gene flow as demonstrated by Pang and Zhang (2024) raises serious concerns about the *f*-branch approach, which relies on those methods.

Fifth, incomplete taxon sampling or taxon exclusion in triplet methods may seriously reduce the information content in the data and even cause unidentifiability issues. A gene flow event between non-sister lineages on the full phylogeny may become an event between sister lineages on the triplet tree and thus unidentifiable by summary methods. For example, the *v→ u* and *z→ w* gene flow in Fig. 1a is between nonsister species but becomes between sisters in the *ADE* triplet. Similarly use of a single sequence per species may create unidentifiability problems (Degnan 2018; Jiao et al. 2021; Yang and Flouri 2022). For example, the direction of gene flow between sister lineages is unidentifiable when only one sequence is sampled from each species, but is identifiable when multiple samples per species are used (Yang and Flouri 2022, Fig. 10). Even when the model is identifiable, including multiple samples per species (in particular, from the recipient population) may boost the information concerning gene flow significantly.

In this study we consider both the presence of ghost lineages and the exclusion of lineages due to the restrictions of the triplet methods as instances of the general problem of incomplete taxon sampling, and study its impacts on inference of gene flow. Besides summary methods, we use the Bayesian method as implemented in the program bpp (Flouri et al. 2018, 2020, 2023). This includes efficient implementations of two classes of models of gene flow in the multispecies coalescent (MSC) framework: the MSC-introgression (MSC-I) model which assumes discrete gene flow with hybridization/introgression at a time point in the past (Wen and Nakhleh 2018; Zhang et al. 2018; Flouri et al. 2020) and the MSC-migration (MSC-M) model which assumes continuous gene flow over an extended time period (Nielsen and Wakeley 2001; Hey et al. 2018; Flouri et al. 2023).

Our paper is structured as follows. First we consider inference of ghost introgression and describe a Bayesian testing approach to comparing models of gene flow in the case of species triplet, including ghost introgression, inflow, and outflow (Figs. 2b–d), as well as the MSC model of no gene flow (Fig. 2a). This follows Pang and Zhang (2024, fig. 3), who demonstrated that commonly used summary triplet methods are unable to identify those models of gene flow (see also Tricou et al. 2022). We use simulation to assess the performance of the Bayesian approach, in comparison with summary triplet methods such as *D* (Green et al. 2010), HyDe(Blischak et al. 2018), SNaQ (Solis-Lemus and Ane 2016) and PhyloNet/MPL (Yu and Nakhleh 2015). We note that in this setting the true model (Figs. 2a–d) is always a special case of the general model (Fig. 2e) and involves at most one gene-flow event. The case of ghost introgression is very complex, and we leave it to the future to evaluate more complex scenarios, for example, those in which gene-flow events are misspecified or multiple ghost lineages are involved. Second, we assess the impacts of the changing population size on the species tree due to incomplete taxon sampling on inference of gene flow. We study the estimation of the population size parameter *θ* for one population when *θ* actually changes over time. We conduct an asymptotic analysis considering the estimate using only two sequences per locus when the number of loci approaches infinity, and use simulation to study the case of multiple sequences per locus. We then study bpp estimation of the introgression probability between two sister species, and the performance of both bpp and summary methods in triplet data. We simulate data on a model species tree for six species to compare bpp and triplet methods for estimating the rate of gene flow and evaluate the *f*-branch approach for summarizing triplet results. Overall the *f*-branch approach appears to be effective for generating hypotheses of gene flow that can be tested using rigorous methods.

**Figure 2.**
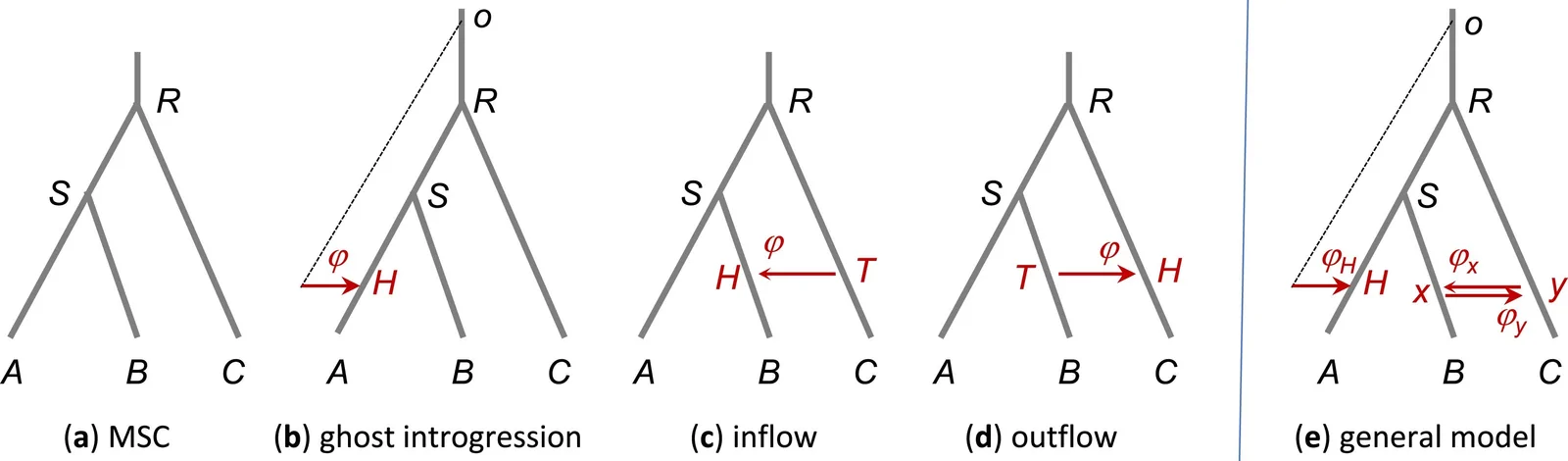
Four MSC-I models used to simulate data: (a) MSC with no gene flow, (b) ghost introgression, (c) inflow from *C → B* (or *y→ x*), and (d) outflow from *B → C* (or *x→ y*). The data are then analyzed using bpp under the full model (e) to select the models of panels a–d. The data are also analyzed using summary methods HyDe and SNaQ, by including one (for HyDe) or two (for SNaQ) distant outgroup species.

## Materials and Methods

### Comparison of Models of Gene Flow for a Species Triplet and Inference of Ghost Introgression

To compare the four models of gene flow of Figs. 2a–d: MSC with no gene flow, ghost introgression, inflow, and outflow, we implement a Bayesian testing approach. We conduct a Markov chain Monte Carlo (MCMC) analysis under an MSC-I model that is more general than and includes all four models of Figs. 2a–d as special cases. The general model involves three introgression probabilities, $\varphi _H, \varphi _x$, and $\varphi _y$, for ghost introgression, inflow, and outflow, respectively (Fig. 2e). We then use the MCMC sample under the general model to test whether each introgression probability is different from the null value of 0. As the hypotheses being tested are nested, the Bayes factor is given by the Savage–Dickey density ratio (Ji et al. 2023). In other words, the Bayes factor in support of the alternative hypothesis of gene flow ($H_1: \varphi >0$) against the null hypothesis of no gene flow ($H_0: \varphi =\varphi _0=0$) is


(1) \begin{eqnarray*}
B_{10} = \frac{\pi (\varphi _0)}{\pi (\varphi _0\mid \boldsymbol x)} = \frac{\pi (\varphi _0)\epsilon }{\pi (\varphi _0\mid \boldsymbol x)\epsilon } \approx \frac{\mathbb {P}(\varphi < \epsilon )}{\mathbb {P}(\varphi < \epsilon \mid \boldsymbol x)},
\end{eqnarray*}

where $\pi (\varphi _0)$ and $\pi (\varphi _0 | \boldsymbol x)$ are the prior and posterior probability densities of *φ* under $H_1$, evaluated at the null value $\varphi _0=0$ (Ji et al. 2023), and where the small value *ε* is fixed at $\epsilon =0.01\sigma = 0.00289$, with $\sigma = 1/\sqrt{12}$ to be the standard deviation of the uniform prior $\varphi \sim U(0,1)$. In practice the prior probability $\mathbb {P}(\varphi < \epsilon ) = \epsilon$ while the posterior probability $\mathbb {P}(\varphi < \epsilon | \boldsymbol x)$ is estimated by the proportion of MCMC samples in which $\varphi < \epsilon$. This S-D approach requires only one run of the MCMC algorithm under the general model of Fig. 2e and is a few hundred times more efficient computationally than calculation of marginal likelihood values under various models (including the four of Figs. 2a–d) using thermodynamic integration combined with Gaussian quadrature, implemented in bpp (Gelman and Meng 1998; Lartillot and Philippe 2006; Rannala and Yang 2017) and used by Pang and Zhang (2024).

We simulated datasets under each of the four models of Figs. 2a–d. The values of parameters used were $\tau _R = 2\theta , \tau _S = \theta , \tau _H = \tau _y = 0.5\theta$, with *θ = 0.01*, while in **b**, $\tau _o = 3\theta$. Note that time is scaled by mutations so that both *τ* and *θ* are measured in units of expected number of mutations per site, and one coalescent time unit is *θ /2* (i.e., *2N* generations for a diploid species with population size *N*). The introgression probability used was *φ =0.2* in **b**–**d**. There were two data sizes, with *L=250* or 1000 loci, with *S=4* sequences per species per locus, and with the sequence length to be 500 sites. The number of replicates was 100. Each dataset was generated using the simulate option in bpp (Flouri et al. 2018; Yang 2015), which generates the gene tree (both tree topology and branch lengths) for each locus, and then simulates the sequence alignment by evolving sequences on the tree under the JC mutation model (Jukes and Cantor 1969).

Each dataset was analyzed under the general model of Fig. 2e using bpp to test the introgression probabilities ($\varphi _H, \varphi _x$, and $\varphi _y$). The JC mutation model was assumed. A gamma prior was assigned on the population size parameter, $\theta \sim$ G(*2, 200*) with the prior mean 0.01. The same population size was assumed for a branch before and after introgression (using the thetamodel = linked-msci option in bpp); for example, *Sx* and *xB* on the species tree (Fig. 2e) were considered one branch and assigned the same *θ*. The age of the root of the species tree was assigned another gamma prior, $\tau _o \sim$ G(*2, 67*) with mean 0.03 (Fig. 2e) while the other node ages have a uniform-Dirichlet distribution (Yang and Rannala 2010, equation 2). The shape parameter *α = 2* is used so that the gamma prior is diffuse. Datasets simulated in this paper were all large and the prior did not have important effects.

We used a burn-in of $4\times 10^4$ iterations, after which we took $10^5$ samples sampling every 2 iterations. Running time for each dataset was 1.5hrs using one thread.

The simulated data were also analyzed using several summary methods including HyDe, implemented using the script run_hyde.py from https://github.com/pblischak/HyDe (Blischak et al. 2018), and SNaQ, implemented in the PhyloNetworks package (Solis-Lemus and Ane 2016), with gene trees for loci inferred using RAxML with default settings (Stamatakis 2014). SNaQ is very similar to PhyloNet/MPL (Yu and Nakhleh 2015). HyDe uses site-pattern counts pooled across all loci, and is calculated using a concatenated alignment. As HyDe requires at least four taxa, a distant outgroup species at the divergence time $\tau _{o_1} = 4\theta$ was included when data were simulated under the models of Figs. 2a–d. SNaQ requires at least five taxa, so we included two distant outgroups at divergence times $\tau _{o_1} = 4\theta$, $\tau _{o_2} = 5\theta$. Only one sequence per species per locus was used for the summary methods.

### One Species: Estimation of *θ* for one Species Assuming a Constant *θ* when *θ* Changes Over Time

We develop an asymptotic theory for estimation of *θ* for one species using *S=2* sampled sequences (of *n = ∞* or 500 sites) when the number of loci $L \rightarrow \infty$. The population size or *θ* is variable, being $\theta _0$ and $\theta _1$ over two time periods (*0, τ*) and ($\tau , \infty$), respectively, but is assumed to be constant when the data are analyzed (Figs. 3a and b). The limiting value of the maximum likelihood estimate (MLE), $\theta _*$, is analytically tractable (see the Results section for the theory). We calculated $\theta _*$ as a function of *τ* for two scenarios: (a) $\theta _0=0.01, \theta _1=0.001$ and (b) $\theta _0=0.001, \theta _1=0.01$.

**Figure 3.**
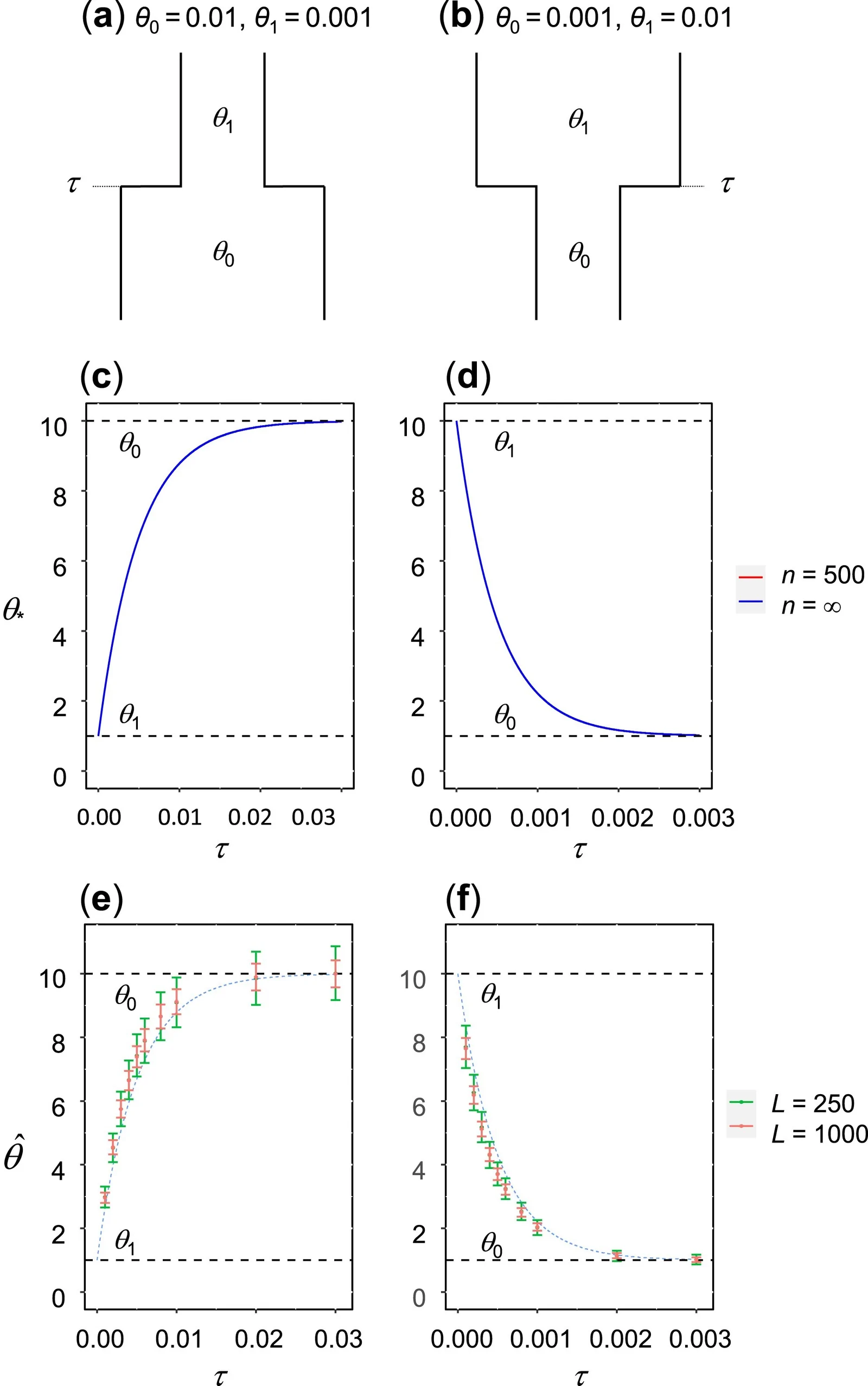
(a, b) Demographic models of population size change, with $\theta _0$ and $\theta _1$ over the time periods (*0, τ*) and ($\tau , \infty$), respectively. In (a), $\theta _0 = 0.01$ and $\theta _1 = 0.001$ while in (b), $\theta _0 = 0.001$ and $\theta _1 = 0.01$. (c, d) Limiting estimates of *θ* under the model of constant size ($\theta _*$ of Equations (6) and (13), $\times 10^3$) when data of infinitely many loci (*L=∞*), each with two sequences of *n = ∞* or 500 sites, are generated under the two-*θ* models of a and b. Estimates for *n = ∞* and 500 are indistinguishable. (e, f) Average posterior means and the 95% HPD CIs for *θ* from bpp analysis of finite data of *L = 250* or 1000 loci, with *S=4* sequences per locus, simulated under the models of panels a and b. Blue dashed lines represent the asymptotic estimates from panels c and d.

We then used simulation to examine Bayesian estimation of *θ* using more than two sequences per locus but with a finite number of loci. We used *S = 4* sequences and *L = 250* or 1000 loci, with the sequence length to be *n = 500* sites. The number of replicates was 100. Each dataset was analyzed using bpp assuming a constant *θ*. The JC mutation model was assumed. A gamma prior was assigned, $\theta \sim$ G(*2, 200*) with the prior mean 0.01 for (a) and $\theta \sim$ G(*2, 2000*) with the mean 0.001 for (b) (Figs. 3a and b). We used a burn-in of $4\times 10^4$ iterations, after which we took $10^5$ samples sampling every 2 iterations. Running time for each analysis was 0.5 hrs using one thread.

### Two Species: Estimation of the Rate of Gene Flow Between Sister Species Using bpp

We simulated data under the MSC-I and MSC-M models of Figs. 4a and b, with gene flow between two sister species, and analyzed the data under the MSC-I model (Fig. 4a). Species represented by thin branches had the population size $\theta _0 = 0.001$ while those for thick branches had a large size $\theta _1 = 0.01$. Note that in this study, the terms species and population are used interchangeably. Species split times were $\tau _R = 10\theta _0$, $\tau _S = 2\theta _0$, $\tau _T = \theta _0$, while introgression time was $\tau _x = \tau _y = 1.5\theta _0$. We used eleven introgression probabilities in the MSC-I model (*φ =* 0, 0.01, 0.05, 0.1, 0.2, 0.3, 0.4, 0.5, 0.6, 0.7, 0.8) and nine migration rates in MSC-M (*M =* 0, 0.01, 0.03, 0.05, 0.1, 0.2, 0.3, 0.4, 0.5). Note that in the MSC-M model, the population migration rate, $M_{xy} = m_{xy}N_y$, is the expected number of migrants from *x* to *y* per generation, with $m_{xy}$ to be the proportion of migrants in population *y* from *x*.

**Figure 4.**
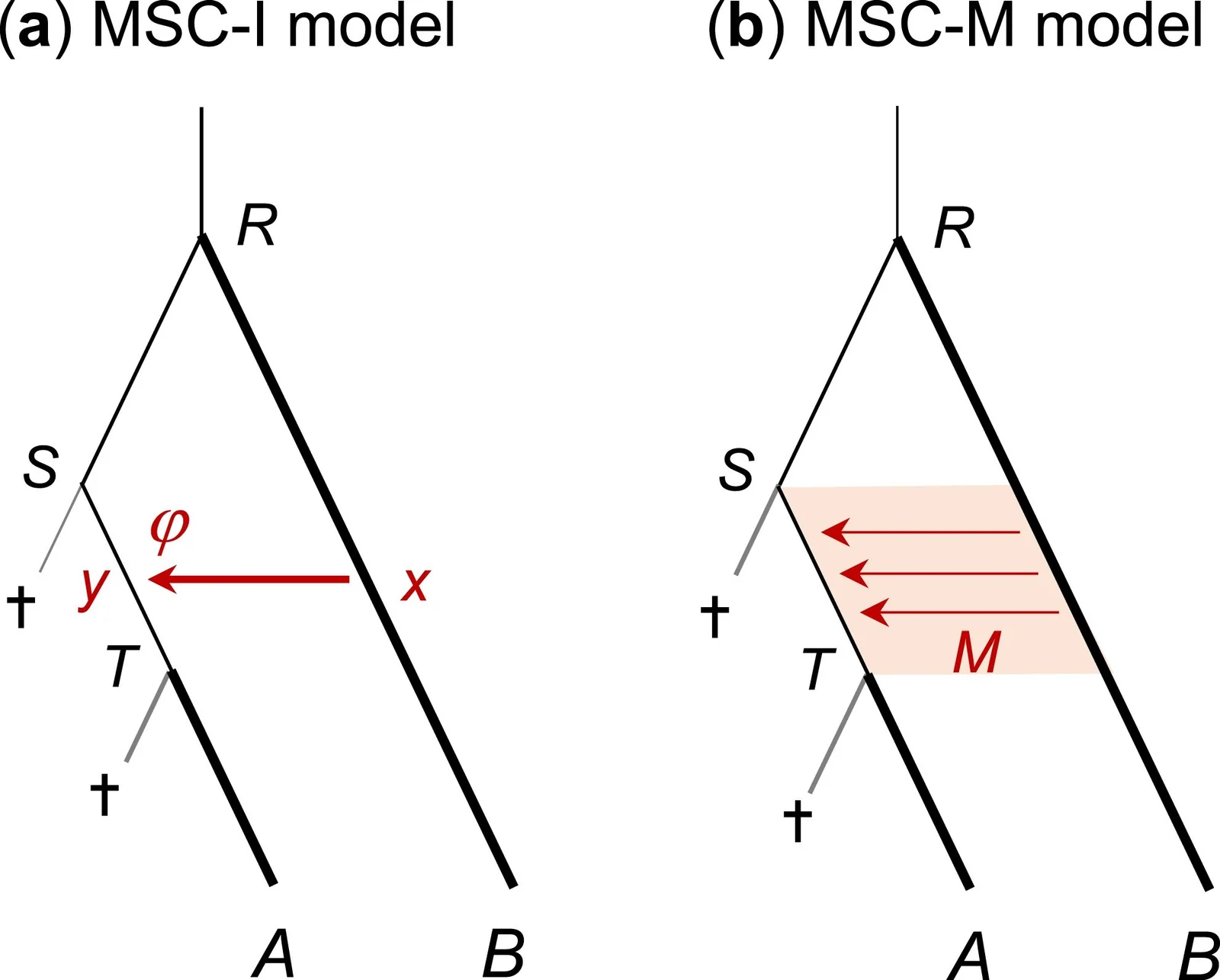
(a) Introgression (MSC-I) and (b) migration (MSC-M) models used to simulate sequence data. Branch thickness represents population size, with $\theta _0 = 0.001$ and $\theta _1 = 0.01$ for thin and thick branches, respectively. Nodes *S* and *T* are created via extinct ghost species to specify different *θ*s for different segments of the branch *RA*, but no sequences were generated for the ghost species.

We generated 100 replicate datasets. Each consisted of *L = 250* or 1000 loci, with *S = 4* sequences per species per locus, and with the sequence length to be *n=500* sites.

Each replicate dataset was analyzed under the MSC-I model (Fig. 4a) using bpp, with the correct donor and recipient populations identified. The root age was assigned the gamma prior $\tau _R \sim$ G(2, 200) with prior mean 0.01, and population sizes were assigned the prior $\theta \sim$ G(*2, 2000*) with mean 0.001. The same population size parameter was assumed for a branch before and after introgression. The introgression probability was assigned the uniform prior, $\varphi \sim$ beta(*1, 1*). We used a burn-in of $4\times 10^4$ iterations, after which we took $10^5$ samples sampling every 2 iterations. Running time was *∼ 1* hour using four threads.

As a reference for comparison, we found it useful to simulate another set of data assuming the same population size for all species (*θ = 0.001*), and analyze the data assuming one population size for the whole tree (using the thetamodel = linked-all option in bpp). The other settings were the same as above for simulation and analysis of data assuming different *θ*s. With one *θ* for all, the issue of incomplete taxon sampling does not arise, and the analysis model is correctly specified.

### Four Species: Estimation of the Rate of Gene Flow using bpp and Summary Methods

The MSC-I and MSC-M models for four species of Figs. 5a and b were used to simulate data, which were analyzed using both bpp and summary methods. As in the case of two species, thin and thick branches on the tree had different population sizes ($\theta _0 = 0.001$ and $\theta _1 = 0.01$, respectively), and an unsampled ghost species was used to break the branch *TB* into two segments with different population sizes. Species split times were $\tau _R = 4\theta _0$, $\tau _S = 3\theta _0$, $\tau _T = 2\theta _0$, $\tau _U = \theta _0$, while introgression time was $\tau _x = \tau _y = 1.5\theta _0$. As in the case of two species, each dataset consisted of *L = 250* or 1000 loci, with *S = 4* sequences per species per locus, and with the sequence length to be *n=500* sites. The number of replicates was 100.

**Figure 5.**
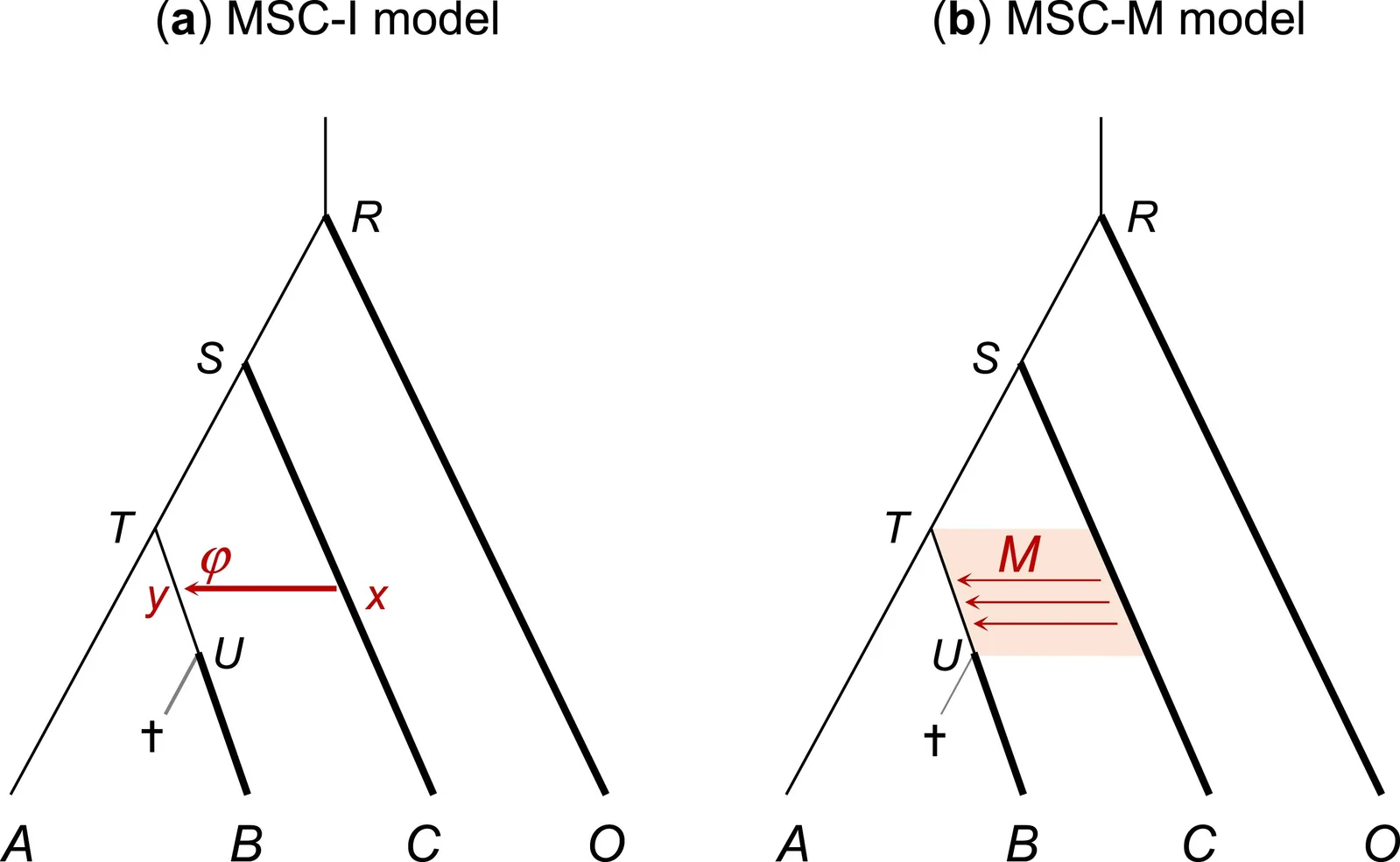
(a) Introgression (MSC-I) and (b) migration (MSC-M) models used to simulate data to compare bpp and summary methods for estimating the rate of gene flow.

Each replicate dataset was analyzed under the MSC-I model of Fig. 5a. Different species on the phylogeny were assigned independent population sizes (*θ*), although the same branch before and after introgression was assigned the same size. The settings for running bpp were the same as in the analysis of the two-species data. The priors were $\theta \sim$ G(2, 400), and $\tau _R \sim$ G(2, 500), and $\varphi \sim$ beta(*1, 1*). We used a burn-in of $4\times 10^4$ iterations, after which we took $10^5$ samples sampling every 2 iterations.

To help interpret the simulation results, we simulated another set of data assuming one population size for all species ($\theta = \theta _0 = 0.001$), and analyze the data assuming one *θ* for all species. All other settings were the same as above.

The simulated data were also analyzed using HyDe and SNaQ (equivalent to PhyloNet/MPL) to infer gene flow and to estimate the introgression probability. Both methods produce point estimates of *φ* only, while bpp provides in addition a measure of uncertainty in posterior CIs. Note that those summary methods were developed under the assumption of one population size (*θ*) for all species on the phylogeny.

### Six Species: Inference of Gene Flow on a Species Phylogeny using bpp and *f*-Branch

We simulated sequence data under the MSC-I model for six species of Fig. 1a and analyzed the data using bpp and the *f*-branch method based on species triplets. We used $\theta _0 = 0.001$ for thin branches and $\theta _1 = 0.01$ for thick branches on the phylogeny. Species split times were $\tau _R = 5\theta _0$, $\tau _S = 4\theta _0$, $\tau _T = 3\theta _0$, $\tau _U = 2\theta _0$, $\tau _V = \theta _0$, $\tau _W = \frac{1}{2}\theta _0$, while introgression times were $\tau _x = \tau _y = 2.5\theta _0$, $\tau _u = \tau _v = 1.5\theta _0$, and $\tau _z = \tau _w = 0.5\theta _0$. The introgression probabilities were $\varphi _y = 0.3, \varphi _u = 0.2$ and $\varphi _w = 0.1$. As in the case of two or four species, we used *L = 250* or 1000 loci, with *S = 4* sequences per species per locus, and with the sequence length to be *n=500*. The number of replicates was 100.

We used bpp to analyse the data under the MSC-I model (Fig. 1a). The same settings were used as above. The priors were $\theta \sim$ G(*2, 400*), and $\tau _R \sim$ G(*2, 400*), and $\varphi \sim$ beta(*1, 1*). We used a burn-in of $4\times 10^4$ iterations, after which we took $10^5$ samples sampling every 2 iterations. Running time for analyzing each dataset was *∼ 1.5* hrs using 4 threads.

We also conducted the Bayesian test of gene flow, using the MCMC sample collected under the MSC-I model to calculate the Bayes factor via the S-D density ratio (Ji et al. 2023).

We also simulated a set of data assuming one population size for all species (*θ =0.001*), and analyzed the data using bpp assuming one *θ* for all species. The other settings were the same as in the analysis of data simulated under different *θ*s.

The simulated data were also analyzed using the *f*-branch approach, implemented in the Dsuite software (Malinsky et al. 2021). The correct species tree of Fig. 1a was provided. The approach first analyzes all triplets, using a triplet method such as $f_4$ (Patterson et al. 2012) and HyDe(Blischak et al. 2018), and then assembles the triplet estimates by attempting to move introgression events onto ancestral branches (Malinsky et al. 2018, 2021).

## Results and Discussion

### Comparison of Models of Gene Flow for a Species Triplet and Inference of Ghost Introgression

Here we follow the setup of Pang and Zhang (2024) to compare several models of gene flow on a species triplet: ghost introgression, inflow, and outflow (Figs. 2b–d), using a computationally efficient approach to calculating the Bayes factor based on the Savage–Dickey density ratio (Equation 1). We simulated multilocus datasets under the models of Figs. 2a–d, and fitted the general model of Fig. 2e to test for the presence of introgression events. When the true model is the MSC model with no gene flow (Fig. 2a), any inferred gene-flow event will be a false positive. The results are summarized in Table 1.

**Table 1.** Power of Bayesian and summary methods to detect introgression, measured as percentages of replicate datasets in which the test supports each of the three introgression events in the general model of Fig. 2e in datasets simulated under the four models of Figs. 2a–d

True model	(a) MSC			(b) Ghost			(c) Inflow			(d) Outflow		
Inferred model	$\varphi _H$	$\varphi _x$	$\varphi _y$	$\varphi _H$	$\varphi _x$	$\varphi _y$	$\varphi _H$	$\varphi _x$	$\varphi _y$	$\varphi _H$	$\varphi _x$	$\varphi _y$
** *L = 250* loci**												
$0 < B_{10} < 0.0101$ (very strong rejection)	**40**	**94**	**95**	0	**97**	**100**	**33**	0	**90**	**41**	**77**	0
$0.0101 < B_{10}<0.0526$ (strong rejection)	**50**	**6**	**5**	0	**3**	**0**	**55**	0	**6**	**51**	**20**	0
$0.0526 < B_{10} < 1$ (rejection)	9	0	0	0	0	0	11	0	4	6	3	0
$1 < B_{10} < 19$ (support)	1	0	0	0	0	0	1	0	0	2	0	0
$19 < B_{10} < 99$ (strong support)	0	0	0	0	0	0	0	0	0	0	0	0
$99 < B_{10} < \infty$ (very strong support)	0	0	0	**100**	0	0	0	**100**	0	0	0	**100**
*Summary methods*												
HyDe (or *D*)	0	13	0	0	100	0	0	**100**	0	0	100	0
SNaQ (or PhyloNet/MPL)	2	42	5	**95**	5	0	30	**28**	41	17	11	**72**
** *L = 1000* loci**												
$0 < B_{10} < 0.0101$ (very strong rejection)	**82**	**100**	**100**	0	**100**	**100**	**84**	0	**100**	**73**	**97**	0
$0.0101 < B_{10}<0.0526$ (strong rejection)	**17**	0	0	0	0	0	**22**	0	0	**13**	**3**	0
$0.0526 < B_{10} < 1$ (rejection)	1	0	0	0	0	0	4	0	0	4	0	0
$1 < B_{10} < 19$ (support)	0	0	0	0	0	0	0	0	0	1	0	0
$19 < B_{10} < 99$ (strong support)	0	0	0	0	0	0	0	0	0	0	0	0
$99 < B_{10} < \infty$ (very strong support)	0	0	0	**100**	0	0	0	**100**	0	0	0	**100**
*Summary methods*												
HyDe (or *D*)	0	12	0	0	100	0	0	**100**	0	0	100	0
SNaQ (or PhyloNet/MPL)	2	43	5	**100**	0	0	15	**50**	35	6	8	**86**

Note.— Bayesian test is based on the Bayes factor, $B_{10}$, calculated using the S-D density ratio with $\epsilon _\varphi = 0.00289$. The cutoffs, $B_{10}= 0.0101, 0.0526, 1$, 19, and 99, correspond to the posterior probability for the model of gene flow $\mathbb {P}(H_1|\boldsymbol x) = 0.01, 0.05, 0.5, 0.95$, and 0.99, respectively. HyDe (or *D*) assumes inflow only, and the support for inflow is significant when the *p*-value is *< 5%*. SNaQ (equivalent to PhyloNet/MPL in the case of four species) ranks the three introgression models by the pseudo-likelihood based on the counts of gene tree topologies. Strong rejection of a gene-flow event (which represents the alternative hypothesis $H_1$) is possible with the Bayesian test but not possible with triplet methods. Strong support for true gene-flow events (and, in the case of the Bayesian test, strong rejection of non-existent gene-flow events) are highlighted in bold. Each column sums to 1 for the Bayesian test, while each row sums to 1 for triplet methods under models with gene flow.

Using bpp, we found strong support for true introgression events and often strong rejection of nonexistent introgression events (Table 1). For example, when data were simulated under MSC with no gene flow, bpp never supported any of the three gene-flow events in any of the datasets. Instead, in each of the large datasets of *L=1000* loci, all three gene-flow events were strongly rejected. The signal of no gene flow was weaker in small datasets of *L=250* loci at the shallow divergence, but even under this scenario, all three gene-flow events were correctly rejected in $\ge 90\%$ of datasets (Table 1 a MSC). Note that strong rejection of the alternative hypothesis ($H_1$) is possible in the Bayesian test.

Similarly in small datasets (*L=250* loci) simulated with ghost introgression, the correct model of ghost introgression ($\varphi _H$) was very strongly supported (with $B_{10} > 99$) in 100% of datasets, while inflow ($\varphi _x$) was very strongly rejected in 97% of datasets (with $B_{10} < 0.0101$), and outflow ($\varphi _y$) was very strongly rejected in 100% of them. The support for the true gene-flow event ($\varphi _H$) and rejection for the nonexistent gene-flow events ($\varphi _x, \varphi _y$) was even more resolute in large datasets of *L=1000* loci (Table 1 b Ghost).

Similarly the correct introgression model was strongly supported in 100% of datasets simulated under the inflow or outflow models, while nonexistent introgression in the opposite direction (or from the ghost species) was strongly rejected (Table 1 c inflow and 1 d outflow). The result is consistent with the previous finding that use of the bidirectional introgression model allows the Bayesian approach to identify the direction of gene flow (Thawornwattana et al. 2023).

The simulated datasets were also analyzed using the summary methods HyDe and SNaQ. HyDe (or the *D*-statistic) assumes inflow only, and provides significant support for inflow when the *p*-value is *< 5%*. When there is gene flow in the true model (Figs. 2b–d), HyDe supported inflow in every dataset, irrespective of the true model, with the false positive rate to be 100% under the ghost-introgression and outflow models, and with the power to be 100% under the inflow model. When the MSC model of no gene flow was used in simulation, HyDe detected significant evidence for *C→ B* (i.e., $\varphi _{y\rightarrow x}$) inflow in 12% of datasets (Table 1 a) and *C→ A* inflow in 13% of datasets, with the false positive rate to be 25%. Note that with no gene flow, the roles of *A* and *B* are equivalent, and the error rate of detecting *C→ B* gene flow equals the rate of detecting *C→ A* gene flow. The high false positive rate of HyDe here may be surprising (as the MSC model of no gene flow is the correct null hypothesis for the test), and appears to be due to the fact that in our simulation sites within the same locus share the same genealogical history whereas all sites are assumed to be independent in the HyDe test, and the incorrect assumption leads to inflated false positives. Consistent with this interpretation, Pang and Zhang (2024, Fig. S1) noted that use of a block-jackknife approach to estimate the *p*-value leads to considerably reduced false positives for HyDe.

SNaQ is equivalent to PhyloNet/MPL in the case of four species, and ranks the three introgression models by the pseudo-likelihood based on the counts of gene tree topologies. It does not provide significance values, so the results are not directly comparable with those of bpp. For simulation under MSC with no gene flow, SNaQ did not select the true model of no gene flow and had a false positive rate of $\sim 100\%$. Table 1 a showed that SNaQ detected the three gene-flow events of Figs. 2b–d in 50% of datasets. In the other half of datasets, it detected other gene-flow events, with the roles of *A* and *B* exchanged. In simulations involving gene flow, SNaQ appeared to favor ghost introgression. When the true model was ghost introgression, the method had high power, inferring the true model in 95% of datasets of 250 loci (with a false positive rate of 5%). However, when the true model was inflow or outflow, the method also detected ghost introgression in 30% or 17% of the datasets, respectively (with high false-positive error rates of 71% or 28%). Overall SNaQ selected the correct model in 95%, 28%, and 72% of datasets, when the true model was ghost introgression, inflow, and outflow, respectively. However, the results were noted to depend on the divergence times for the two outgroups, and at $\tau _{o_1} = 7\theta , \tau _{o_2} = 11\theta$, the corresponding proportions were 32%, 48%, and 17%. As the models under comparison are unidentifiable, the results depend on idiosyncrasies of software implementation. The results are consistent with Pang and Zhang (2024, Fig. 3).

In summary, when there is gene flow, HyDe and SNaQ (or PhyloNet/MPL) cannot distinguish among the different models of gene flow: ghost introgression, inflow, and outflow (Figs. 2b–d). Those results are consistent with Pang and Zhang (2024, Fig. 3). When there is no gene flow, HyDe and SNaQ show high false positives of inferring gene flow (25% and 100%, respectively; Table 1). In contrast, the Bayesian test showed excellent statistical performance, strongly supporting true gene-flow events and strongly rejecting nonexisting gene flow events (Table 1).

### One Species: Estimation of *θ* for One Species When Population Size Changes Over Time

As the introgression probability is defined as the expected proportion of migrants in the recipient population, the changing size of the recipient population (possibly due to incomplete taxon sampling) may affect our definition or estimation of the rate (or magnitude) of gene flow. Here we use mathematical analysis and computer simulation to examine the estimate of the population size for a species when the size actually changes over time.

Consider a change-point model, with *θ* for a single population changing over time, being $\theta _0$ and $\theta _1$ over two time periods (*0, τ*) and ($\tau , \infty$), respectively (Fig. 3a). We estimate the population size parameter (*θ*), assuming a constant population size. The case of two sequences per locus is tractable analytically.

Let *θ (t)* be the population size time *t* ago. The density for the coalescent time (*t*) between two sequences is then


(2) \begin{eqnarray*}
f(t) = \frac{2}{\theta (t)} \exp \biggl \lbrace - \int _0^t \frac{2}{\theta (u)} \, \mathrm{d}u \biggr \rbrace
\end{eqnarray*}

Slatkin and Hudson 1991; Griffiths and Tavaré 1994). With the two-*θ* model of Fig. 3a, this simplifies to


(3) \begin{eqnarray*}
f(t;\boldsymbol \theta ) = \left\lbrace \begin{array}{@{}l@{\quad }l@{}}\frac{2}{\theta _0}\operatorname{\mathrm{e}}^{-\frac{2}{\theta _0}t} , & \mathrm{if } t<\tau , \\
\operatorname{\mathrm{e}}^{-\frac{2}{\theta _0}\tau }\frac{2}{\theta _1}\operatorname{\mathrm{e}}^{-\frac{2}{\theta _1}(t-\tau )}, & \mathrm{otherwise.} \end{array}\right.
\end{eqnarray*}

Suppose coalescent times at *L* loci, $t_i$, *i=1,⋯ ,L*, are available and used to estimate *θ* under the model of a constant population size ($H_0$). The coalescent time has the density


(4) \begin{eqnarray*}
f_0(t; \theta ) = \frac{2}{\theta }\operatorname{\mathrm{e}}^{-\frac{2}{\theta }t} ,
\end{eqnarray*}

giving the likelihood $L_0(\theta ) = \prod _i \frac{2}{\theta }\operatorname{\mathrm{e}}^{-\frac{2}{\theta }t_i}$. The MLE under $H_0$ is simply


(5) \begin{eqnarray*}
\hat{\theta }= 2\bar{t} = 2\sum _i t_i/L.
\end{eqnarray*}

When $L\rightarrow \infty$, the MLE $\hat{\theta }$ approaches the limiting value $\theta _*$, which minimizes the Kullback–Leibler (KL) divergence


(6) \begin{eqnarray*}
D_t = \int _{0}^{\infty }f(t; \boldsymbol \theta )\log \frac{f(t; \boldsymbol \theta )}{f_0(t; \theta )}\, \mathrm{d}t.
\end{eqnarray*}

Note that $\theta _*$ is also the limiting value of the Bayesian estimate (posterior mean) as $L \rightarrow \infty$. It is known as the *best-fitting parameter value* or *pseudo-true parameter value* under $H_0$. In Equation (6), $f(t;\boldsymbol \theta )$ represents the data while $f_0(t;\theta )$ represents the fitting model, and minimization of Equation (6) is equivalent to maximization of the average log likelihood per datum under the fitting model $H_0$,


(7) \begin{eqnarray*}
\begin{aligned} \ell _0(\theta ) &= \int _{0}^{\infty }f(t;\boldsymbol \theta )\log (f_0(t;\theta ))\, \mathrm{d}t \\
&= \int _{0}^{\infty }f(t;\boldsymbol \theta ) \bigl [\log \tfrac{2}{\theta } - \tfrac{2}{\theta }t \bigr ] \, \mathrm{d}t \\
&= \log \tfrac{2}{\theta } - \tfrac{2}{\theta } \operatorname{\mathbb {E}}_{\boldsymbol \theta }(t) , \end{aligned}
\end{eqnarray*}

giving


(8) \begin{eqnarray*}
\begin{aligned} \theta _* &= 2\operatorname{\mathbb {E}}_{\boldsymbol \theta }(t) = 2\int _0^\infty tf(t;\boldsymbol \theta ) \, \mathrm{d}t \\
&= \theta _0 + \operatorname{\mathrm{e}}^{-\frac{2}{\theta _0}\tau }(\theta _1 - \theta _0) . \end{aligned}
\end{eqnarray*}

This is a weighted average of $\theta _0$ and $\theta _1$, and the weight $\operatorname{\mathrm{e}}^{-\frac{2}{\theta _0}\tau }$ for $\theta _1$ is the probability that the coalescence occurs over ($\tau ,\infty$) when the population size is $\theta _1$ (Fig. 3).

Treating coalescent times as data is equivalent to assuming infinitely long sequences. Next we consider finite sequences with *n* sites. We assume the infinite-sites mutation model. The number of mutations (*x*) between two sequences of length *n*, given the coalescent time *t*, has the Poisson distribution


(9) \begin{eqnarray*}
f(x \mid t) = \frac{1}{x!}(2nt)^x \operatorname{\mathrm{e}}^{-2nt} ,
\end{eqnarray*}

where *2nt* is the expected number of mutations between the two sequences. The unconditional probability is an average over the coalescent time,


(10) \begin{eqnarray*}
f(x; \boldsymbol \theta ) &=& \int _{0}^{\infty }f(t)f(x\mid t)\, \mathrm{d}t \\
&=& \frac{1}{x!n\theta _0} \Bigl ( \frac{n\theta _0}{1+n\theta _0}\Bigr )^{x+1} \gamma \Bigl (x+1, \bigl (\tfrac{2}{\theta _0}+2n\bigr )\tau \Bigr ) \\
&&+ \frac{1}{x!n\theta _1} \Bigl ( \frac{n\theta _1}{1+n\theta _1}\Bigr )^{x+1} \operatorname{\mathrm{e}}^{-\bigl ( \frac{2}{\theta _0} - \frac{2}{\theta _1} \bigr )\tau } \\
&&\times \Bigl [ \Gamma (x+1) - \gamma \Bigl (x+1, \bigl (\tfrac{2}{\theta _1}+2n\bigr )\tau \Bigr ) \Bigr ] ,
\end{eqnarray*}

where


(11) \begin{eqnarray*}
\gamma (a,x) = \int _0^x t^{a-1}\operatorname{\mathrm{e}}^{-t} \, \mathrm{d}t
\end{eqnarray*}

is the incomplete gamma function and $\Gamma (a) = \gamma (a,\infty )$ is the gamma function, with *Γ (x+1) = x!* as *x* is a non-negative integer.

Under $H_0$ with a constant *θ*, *x* has the density


(12) \begin{eqnarray*}
\begin{aligned} f_0(x; \theta ) &= \int _0^\infty f_0(t)f(x|t)\, \mathrm{d}t \\
&= \frac{1}{n\theta + 1} \Bigl ( \frac{n\theta }{1+n\theta } \Bigr )^x . \end{aligned}
\end{eqnarray*}

In other words, the number of segregating sites (*x*) has the geometric distribution (Watterson 1975).

Again by minimizing the K-L divergence,


(13) \begin{eqnarray*}
D_x = \sum _{x=0}^n f(x; \boldsymbol \theta ) \log \frac{f(x; \boldsymbol \theta )}{f_0(x; \theta )} ,
\end{eqnarray*}

we obtain the best-fitting parameter value $\theta _*$ under $H_0$, as a function of ($\tau , \theta _0, \theta _1$) in the generating model and the sequence length (*n*). In this case the solution is not analytical. Instead we use the BFGS algorithm implemented in paml (Yang 2007) to find $\theta _*$ to minimize $D_x$ (Equation 13), with Gaussian quadrature used to calculate the 1-D integral numerically, using 64 quadrature points.

The limiting estimate $\theta _*$ is plotted against *τ* in Figs. 3c and d for two sets of population sizes: (a) $\theta _0 = 0.01, \theta _1 = 0.001$ and (b) $\theta _0 = 0.001, \theta _1 = 0.01$. The results for *n = ∞* and *n = 500* are indistinguishable. In the special case of *τ = 0*, we have $\theta _* = \theta _1$ while if $\tau =\infty$, $\theta _* = \theta _0$. When $0 < \tau < \infty$, $\theta _*$ is an average between $\theta _0$ and $\theta _1$, with weights to be the probabilities that the coalescent occurs in the two time periods (Equation 8). At $\tau = \log 2 \times \frac{\theta _0}{2} = 0.35\theta _0$, half of the coalescent events occur before reaching *τ* (that is, with *t<τ*), so that $\theta _* = \frac{\theta _0 + \theta _1}{2}$. At $\tau = 2\theta _0$, 98% ($=\operatorname{\mathrm{e}}^{-4}$) of coalescent events occur before *τ*, so that $\theta _*$ is dominated by $\theta _0$.

We then simulated finite datasets of *S=4* sequences per locus and *L = 250* or 1000 loci, with posterior means and the 95% highest posterior density (HPD) credibility intervals (CIs) shown in Figs. 3e and f. There was more uncertainty in estimates for *L=250* than for *L=1000*, but the means were similar between the two data sizes, which may be expected to be close to the limiting value $\theta _*$ when $L \rightarrow \infty$. While the mean value is a weighted average of $\theta _0$ and $\theta _1$, it is closer to $\theta _0$ than in the case of *S=2* sequences. This is because with more sequences in the sample, a greater proportion of coalescent events occurs in the recent time interval (*0,τ*), so that the estimate $\theta _*$ will be closer to $\theta _0$. Overall, the results for data of four sequences (Figs. 3e and f) were similar to those for two sequences (with either *n = ∞* or *n=500* sites) (Figs. 3c and d).

In summary, when the population size for a species changes over time but is assumed to be constant, our estimate from genomic data will be a weighted average, with the weight to be the proportion of coalescent events (Equation 8). When more sequences are sampled from the species, a greater proportion of coalescent events will occur in the recent past, and the estimated population size will reflect more recent population sizes (i.e., $\theta _*$ is closer to $\theta _0$ for *S=4* than for *S=2* in Figs. 3e and f).

### Two Species: Estimation of the Rate of Gene Flow Between Sister Species

When there is gene flow between two sister lineages (*B→ A* or *x→ y* in Figs. 4a and b), incomplete taxon sampling may cause branches on the phylogeny to be merged into one branch. This causes a re-definition of the recipient population and may affect our estimation of the introgression probability (*φ* in Fig. 4a). For example, in the MSC-I model of Fig. 4a, introgression occurs at time $\tau _x = \tau _y$ when the recipient population has the small size $\theta _0$. When data from the modern species *A* and *B* are analyzed, the three branches *RS*, *ST*, and *TA* (with population sizes $\theta _0, \theta _0, \theta _1$, respectively), are merged into one branch *RSTA* and assigned the same size $\theta _A$. What will be the estimated introgression probability *φ*? Here we address this question by simulation. We used both the MSC-I and MSC-M models of Figs. 4a and b to simulate data and analyzed them under the MSC-I model using bpp. Summary methods considered in this study cannot infer gene flow between sister species and are not used.

As a reference for comparison, we have found it useful to examine the estimation problem when all species on the phylogeny are assumed to have the same size (*θ*). In such cases, incomplete taxon sampling has no effect, the assumed MSC-I model is correct, and the results represent the best-case scenario. We thus discuss those results first (Supplementary Figs. S1a and b), before simulations in which different populations are allowed to have different sizes (Fig. 6).

**Figure 6.**
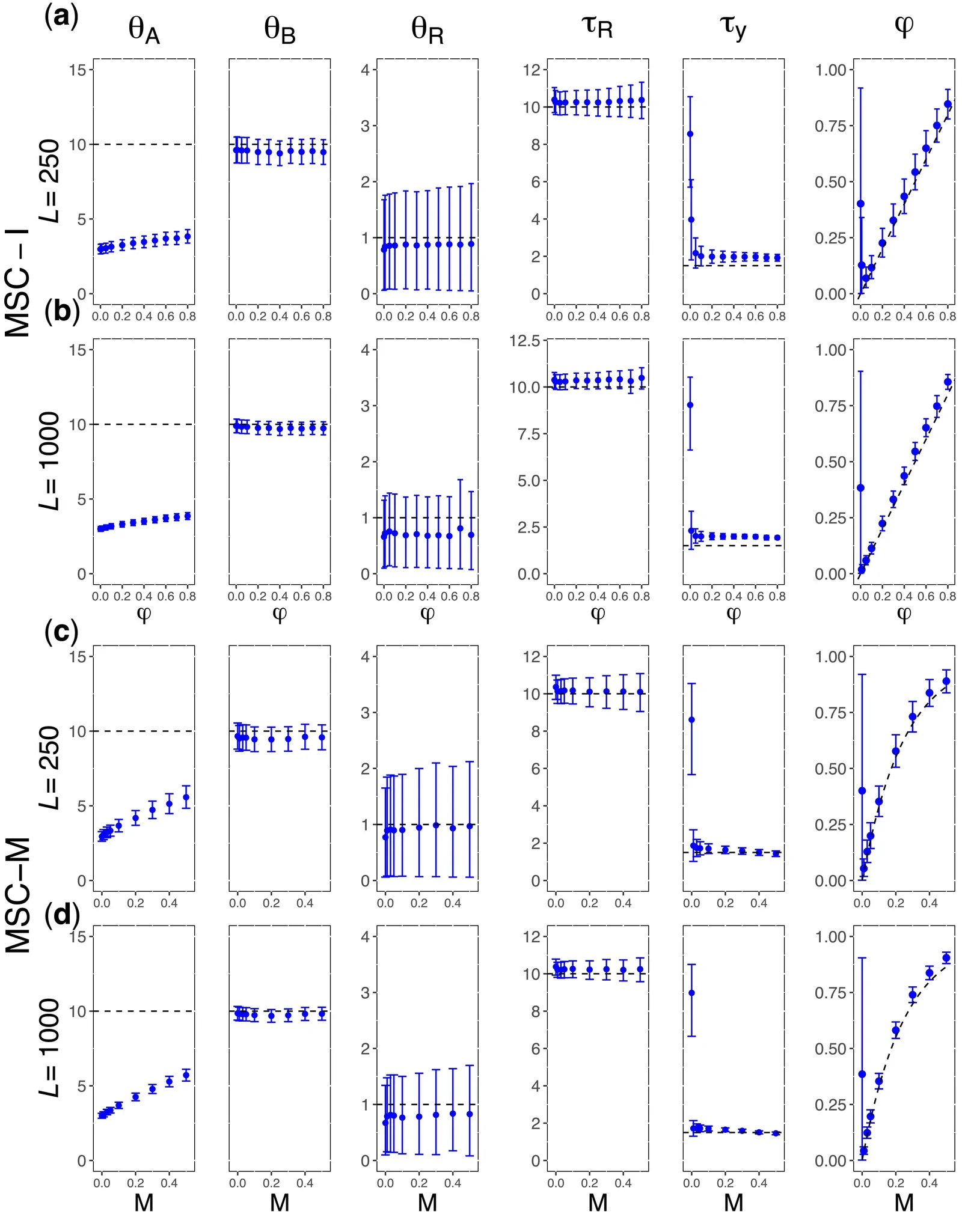
(a, b) Average posterior means and the 95% HPD CIs of parameters when the data are simulated and analyzed under the MSC-I model (Fig. 4a) assuming different *θ*s for species, plotted against the introgression probability (*φ*). (c, d) Parameter estimates under MSC-I when data are simulated under MSC-M (Fig. 4b), plotted against the migration rate (*M*). Estimates of *θ* and *τ* are mutiplied by 1000. Black dashed lines represent the true value in a and b or expected value ($\varphi _0$) in c and d.

#### One population size for all species

When data are simulated and analyzed assuming one population size (Supplementary Figs. S1a and b), *θ* was very precisely and accurately estimated, even at *L=250* loci. Other parameters ($\tau _R, \tau _y, \varphi$) were well estimated as well when the rate of gene flow was not very low. However, when the true rate was very low (*φ = 0* or 0.01), the introgression probability (*φ*) had large uncertainties, with the CI covering almost the whole range (0,1) for the parameter. The other parameters were affected as well.

For data simulated under MSC-M, the mode of gene flow is misspecified but the assumptions about population sizes are all satisfied (Supplementary Figs. S1c and d). The results were strikingly similar to those of Supplementary Figs. S1a and b, where the MSC-I model was used both to simulate and to analyze data. The misspecification of the mode of gene flow had very little impact. Parameter *θ* was very accurately estimated, as are other parameters, except when gene flow was absent (*M=0*). When *M=0*, the species split time, the introgression time, and the introgression probability involved large uncertainties (Supplementary Figs. S1c and d).

Poor estimation of the rate of gene flow at very low rates was noted by Huang et al. (2022, Fig. 3, *M*), where data were simulated under the MSC-M model with migration between sister species and analyzed under the MSC-I model. Here the same pattern is seen whether the MSC-I or MSC-M model is used to generate data. This may reflect the nonstandard features of the MSC-I model of gene flow between sister lineages when the data are generated under the model of no gene flow, discussed by Yang et al. (2026). For example both *φ =0* and *φ =1* (Fig. 4a) correspond to the scenario of no gene flow, which explains the extremely wide CIs for *φ* in Supplementary Fig. S1, *φ*) when data are simulated with no or little gene flow.

When there is continuous migration from populations *x* to *y* over time period $\Delta \tau$, the total amount of gene flow expected under the MSC-M model may be measured by the probability that a sequence from the recipient population *y* is traced back to the hybridizing population *x*, given as


(14) \begin{eqnarray*}
\varphi _0 = 1 - \operatorname{\mathrm{e}}^{-\varpi _{xy}\Delta \tau }
\end{eqnarray*}

where $\varpi _{xy} = m_{xy}/\mu = 4M_{xy}/\theta _y$ is the mutation-scaled migration rate ( Huang et al. 2022, equation 10).

When the migration rate *M* increases, the expected amount of gene flow $\varphi _0$ increases, and the estimated *φ* under the MSC-I model closely tracked the expectation $\varphi _0$, suggesting that the MSC-I model was able to recover nearly all the gene flow that occurred in the data (but note that $\hat{\varphi }$ has large uncertainties when *M ≈ 0*, as discussed above) (Supplementary Figs. S1c and d).

#### Different population sizes with incomplete taxon sampling

Results for data simulated assuming different population sizes for species on the phylogeny (Fig. 4) are summarized in Figs. 6a and b for data simulated under the MSC-I model and in Figs. 6c and d for data simulated under MSC-M. In the simulation models (Figs. 4a and b), the recipient population (branch *RA*) is represented by three segments with different population sizes, a small size $\theta _0 = 0.001$ for *RS* and *ST* and a large size $\theta _1 = 0.01$ for *TA*. When the data were analyzed, one population size was assigned to the whole branch ($\theta _A$ for branch *RA* or *RSTA*).

As in the analysis of data assuming one *θ* for all species, the results were very similar between data simulated under MSC-I and under MSC-M (Figs. 6 a–d), and the misspecification of the mode of gene flow had little impact. Our analysis of the case of one species (Figs. 3a–f) suggests that here the estimated $\theta _A$ may be an average of $\theta _0 = 0.001$ and $\theta _1 = 0.01$ in the true model, with weights corresponding to the proportions of coalescent events that occur in populations *RT* (with $\theta _0 = 0.001$) and *TA* ($\theta _1 = 0.01$). The slight increase of $\hat{\theta }_A$ with the increase of the rate of gene flow (*φ* in MSC-I and *M* in MSC-M) may be explained similarly, as a high *B→ A* rate of gene flow means few *A* lineages enter and coalesce in population *RSY*. Population size $\theta _B$ was unaffected by taxon sampling or gene flow and was well estimated. The species split time and the population size at the root ($\tau _R, \theta _R$) were well estimated but had larger uncertainties than in the case of one *θ* for all populations (Supplementary Fig. S1).

The introgression probability (*φ*) was well estimated except at very low rates of gene flow (*φ =0* and 0.01 in MSC-I and *M=0* in MSC-M, Fig. 6). When the data were generated under MSC-M and analyzed under MSC-I, the estimate $\hat{\varphi }$ was close to the expectation $\varphi _0$ (Figs. 6c and d), suggesting that the MSC-I model recovered almost all gene flow that occurred in the data. The estimates were nearly as good as those for data simulated assuming the same *θ* (Supplementary Fig. S1). This was the case even when the population size for the recipient species ($\theta _A$) was incorrect by a few folds (note that the true size is $\theta _0 = 0.001$ for *RST* and $\theta _1 = 0.01$ for *TA*). The results confirm our expectation that genomic data may be informative about the proportion of immigrants rather than the number of immigrants (see Introduction). For example, when *φ = 0.2*, the estimated ‘expected number of immigrants’ is $\hat{\varphi }\hat{\theta }_A = 0.022 \times 0.0033 \approx 0.00073$ (Fig. 6b, MSC-I, *L=1000*), 3.6 times as large as the true number, $\varphi \theta _0 = 0.2 \times 0.001 = 0.0002$, whereas $\hat{\varphi }= 0.21$ is very close to *φ = 0.2*.

In summary, when the population size for the recipient species of gene flow changes, possibly due to incomplete taxon sampling, genomic data allows accurate estimation of the introgression probability, which is the expected proportion of immigrants in the recipient population. In contrast the expected number of immigrants is not reliably estimated. This may be because the distribution of gene trees depends on the introgression probability, which is the probability that a sequence from the recipient population is traced to the hybridizing population.

### Four Species: Estimation of the Rate of Gene Flow by bpp and Quartet Summary Methods

We use the phylogeny for a species quartet of Fig. 5 with gene flow (inflow) to assess the impact of changing population sizes (possibly due to incomplete taxon sampling) on estimation of the rate of gene flow. Both the MSC-I and MSC-M models (Figs. 5a and b) are used to generate data, which are analyzed under the MSC-I model using bpp. We also apply summary quartet methods HyDe (or *D*) and SNaQ (or PhyloNet/MPL) to the same data. Note that those summary methods assume one population size for all species on the phylogeny.

Again as a reference for comparison, we first discuss the results for data simulated assuming one population size for all species (Supplementary Fig. S2 and Fig. 7).

**Figure 7.**
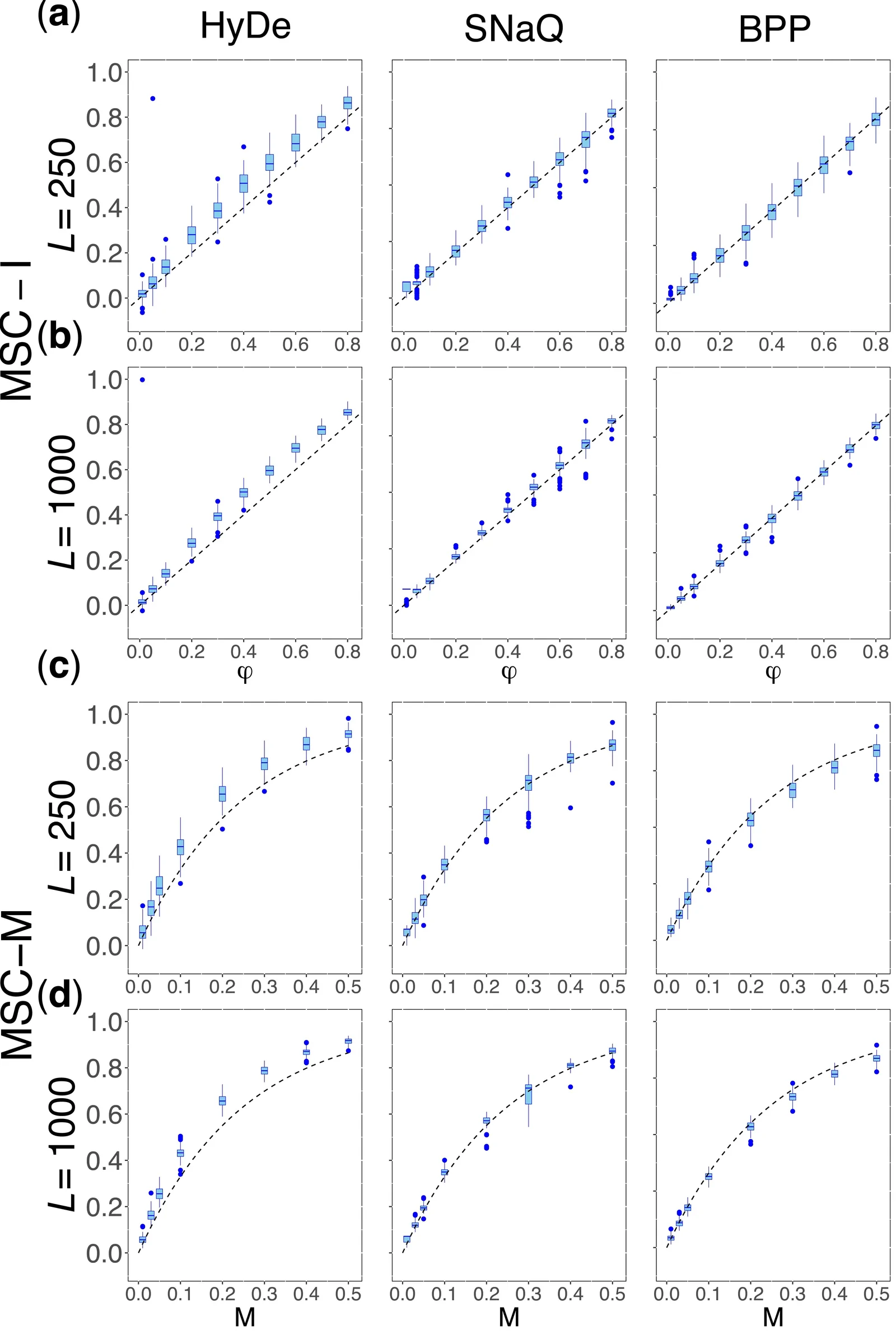
Boxplots of estimates of the introgression probability *φ* using (a) Hyde and (b) SNaQ in analyses of data simulated under the MSC-I and MSC-M models of Figs. 5a and b assuming one *θ* for all species. (c) Results for bpp of Figure S2 are plotted here for comparison. In a boxplot, the bar represents the median, the box the interquartile range (IQR), while the whiskers represent the minimum and maximum defined as the lower and upper quartile plus *1.5 ×* IQR. Note that the average 95% CI for *φ* was shown for bpp in Supplementary Fig. S2 whereas here the IQR based on point estimates is shown.

#### One population size for all species

The results for bpp are summarized in Supplementary Fig. S2. In Supplementary Figs. S2a and b, the MSC-I model was used both to simulate and analyze data, with no model misspecification. The results thus represent the best-case scenario. The posterior means for species split times and introgression times ($\tau _R, \tau _S, \tau _T, \tau _Y$), and the population size (*θ*) were all close to the true values and the CIs were narrow and included the true values. When the true *φ* was very low, estimates of the introgression time $\tau _y$ involved large uncertainties. This can be explained by the rarity of introgression events on the gene trees or in the sequence data: if an event is rare, it will be hard to estimate the time of the event. In contrast, at high *φ*, the split time $\tau _T$ involved large uncertainties. This is apparently due to the negative correlation between $\tau _T$ and *φ*: a high rate of gene flow with an ancient split may fit the data nearly equally well as a low rate of gene flow and recent split. Overall, the introgression probability (*φ*) was well estimated. Asymptotic theory predicts that quadrupling the amount of data reduces the CI by a half, a prediction that roughly holds when we compare the CIs for *L=250* and 1000.

In Supplementary Figs. S2c and d, data were simulated under the MSC-M or secondary-contact model of Fig. 5b and analyzed under the MSC-I model of Fig. 5a, so that the mode of gene flow was misspecified. Species split times and the population size were well estimated, as in Supplementary Figs. S2a and b. The estimated introgression time ($\tau _y$) was less than the average migration time, that is, $\hat{\tau }_y < (\tau _T + \tau _U)/2$. This is because recent migration events on the gene trees close to $\tau _U$ in the data can only be explained by a recent introgression time in the MSC-I model (Huang et al. 2022).

The amount of gene flow predicted in the MSC-M model (Equation 14) gave a close match to estimates of *φ* in MSC-I from bpp (Supplementary Figs. S2c and d), suggesting that the MSC-I model recovered almost all of the gene flow that occurred in the data simulated under the MSC-M model despite the misspecification of the mode of gene flow.

The simulated data were also analyzed using the summary methods HyDe and SNaQ to estimate the introgression probability (Fig. 7). All estimates were well-behaved, although HyDe appeared to overestimate *φ* slightly. The bias may be due to the fact that HyDe assumes a hybrid-speciation model (that is, a symmetrical inflow model with $\tau _T = \tau _y = \tau _x$ in Fig. 5a) whereas in the true model $\tau _T > \tau _x$ (Ji et al. 2023). Note that HyDe and SNaQ do not provide estimates for species split times or introgression time.

#### Different population sizes for species on the phylogeny

Quartet data generated under different population sizes (Figs. 5a and b) were analyzed using bpp in two ways, assuming either different population sizes (Fig. 8) or the same population size (Supplementary Fig. S3).

**Figure 8.**
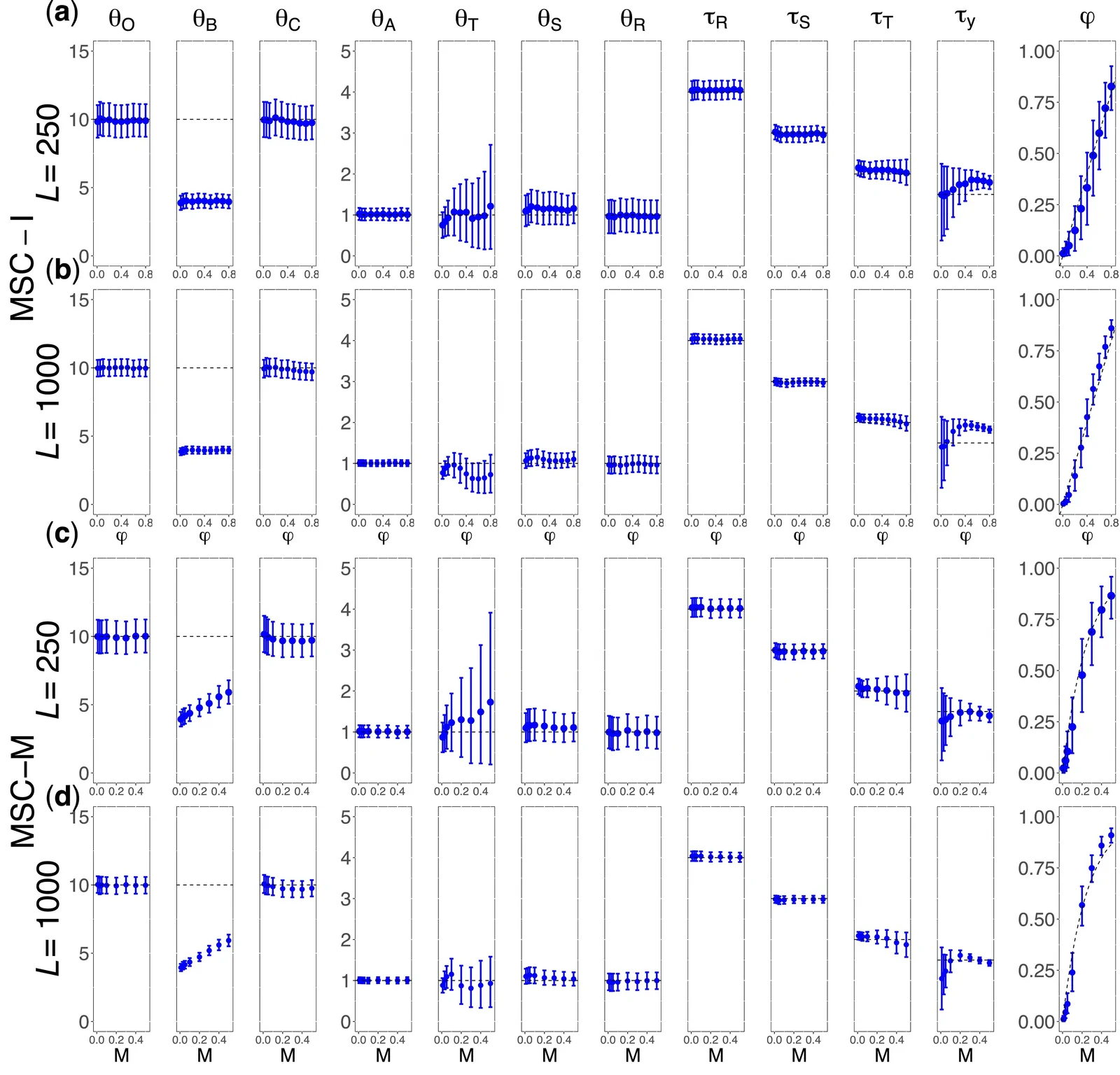
(a, b) Average posterior means and the 95% HPD CIs for parameters when data are simulated and analyzed under the MSC-I model of Fig. 5a assuming different *θ*s for species, plotted against the introgression probability (*φ*). (c, d) Estimates under MSC-I when data are simulated under MSC-M (Fig. 5b), plotted against the migration rate (*M*). Dashed lines represent true parameter values except that in c and d for $\tau _y$ and *φ*, they represent $(\tau _T+\tau _U)/2$ and $\varphi _0$ (Equation 14), respectively.

We first consider bpp analysis assuming different *θ*s (Figs. 8a and b). Note that the presence of a ghost species in the model tree (Fig. 5) means that the recipient population of gene flow is represented by branch *TUB* consisting of segments with different population sizes, so that the model is misspecified even if different *θ*s are assumed for different species in the model. As there are now far more parameters in the model, the estimates, especially population sizes for ancestral species (Figs. 8a and b), had much larger uncertainties than in data simulated assuming one *θ* (Supplementary Fig. S2). Nevertheless, the *θ* and *τ* parameters were well estimated. Species split times ($\tau _R, \tau _S, \tau _T$) appeared to be nearly as well-estimated as in the case of one *θ* for all populations. Similar to Supplementary Fig. S2, $\tau _T$ had larger CIs at high rate of gene flow and $\tau _y$ had larger CIs at low rate of gene flow.

Because branches *TU* and *UB*, which had different population sizes in the true model ($\theta _0 = 0.001$ for *TU* and $\theta _1 = 0.01$ for *UB*, Fig. 5a), are merged into one branch and assigned one population size ($\theta _B$) in the analysis model, the estimated $\theta _B$ is expected to be a weighted average of $\theta _B=0.01$ and $\theta _U=0.001$, depending on whether sequences from *B* coalesce with each other before or after $\tau _U$ (Figs. 8a and b). Despite the poor estimation of $\theta _B$, estimates of *φ* were close to the true values (Figs. 8a and b), although they involved greater uncertainties than in the case of one *θ* for all populations (Supplementary Figs. S2a and b). The pattern mimics the reliable estimation of the introgression probability and the poor estimation of the recipient population size for simulations in the case of two species discussed above (Fig. 6, *φ* & $\theta _B$).

When the data were generated under MSC-M but analyzed under MSC-I (Figs. 8c and d), the results were similar to those obtained from the data simulated under MSC-I (Figs. 8a and b). At higher migration rate ($M = M_{CB}$), the estimated $\theta _B$ was larger. This is apparently because $\theta _B$ is estimated based on coalescent events of *B* sequences along the branch *BUT*, and a high migration rate reduces the proportion of coalescent events along branch *UT* (which had the small population size, Fig. 5b). The estimates of *φ* were close to the expected $\varphi _0$ (Equation 14), suggesting that the amount of gene flow expected under the MSC-M model was nearly correctly recovered by the MSC-I model.

We also analyzed the data of Fig. 8 simulated with different *θ*s using bpp under the MSC-I model assuming one *θ* for all populations (Supplementary Fig. S3). The analysis model was wrong, and parameter estimates had large biases. While there was a positive correlation between the true introgression probability in the MSC-I model and the Bayesian estimates, the match was poor (Supplementary Figs. S3a and b, *φ*). Estimates of introgression time were affected as well (Supplementary Figs. S3a and b, $\tau _y$). When the data were simulated under MSC-M with different *θ*s but analyzed under MSC-I assuming the same *θ*, parameter estimates appeared to be less biased, with the estimated introgression probability $\hat{\varphi }$ being lower than but tracking the expected value $\varphi _0$ (Supplementary Figs. S3c and d).

Overall, bpp analysis was affected far more by the assumptions of equal population size on the species phylogeny than by the mode of gene flow (MSC-I versus MSC-M).

The simulated data of Fig. 8 were also analyzed using the summary methods HyDe and SNaQ (Fig. 9). Note that HyDe and SNaQ were developed under the assumption of one population size for all species on the triplet tree. The estimates of introgression probability produced by HyDe and SNaQ were poor (Fig. 9), somewhat similar to bpp estimates under the assumption of one *θ* for all populations and more different from bpp estimates under the assumption of different *θ*s. The results are in sharp contrast to the accurate estimates of Fig. 7 for data simulated assuming one population size.

**Figure 9.**
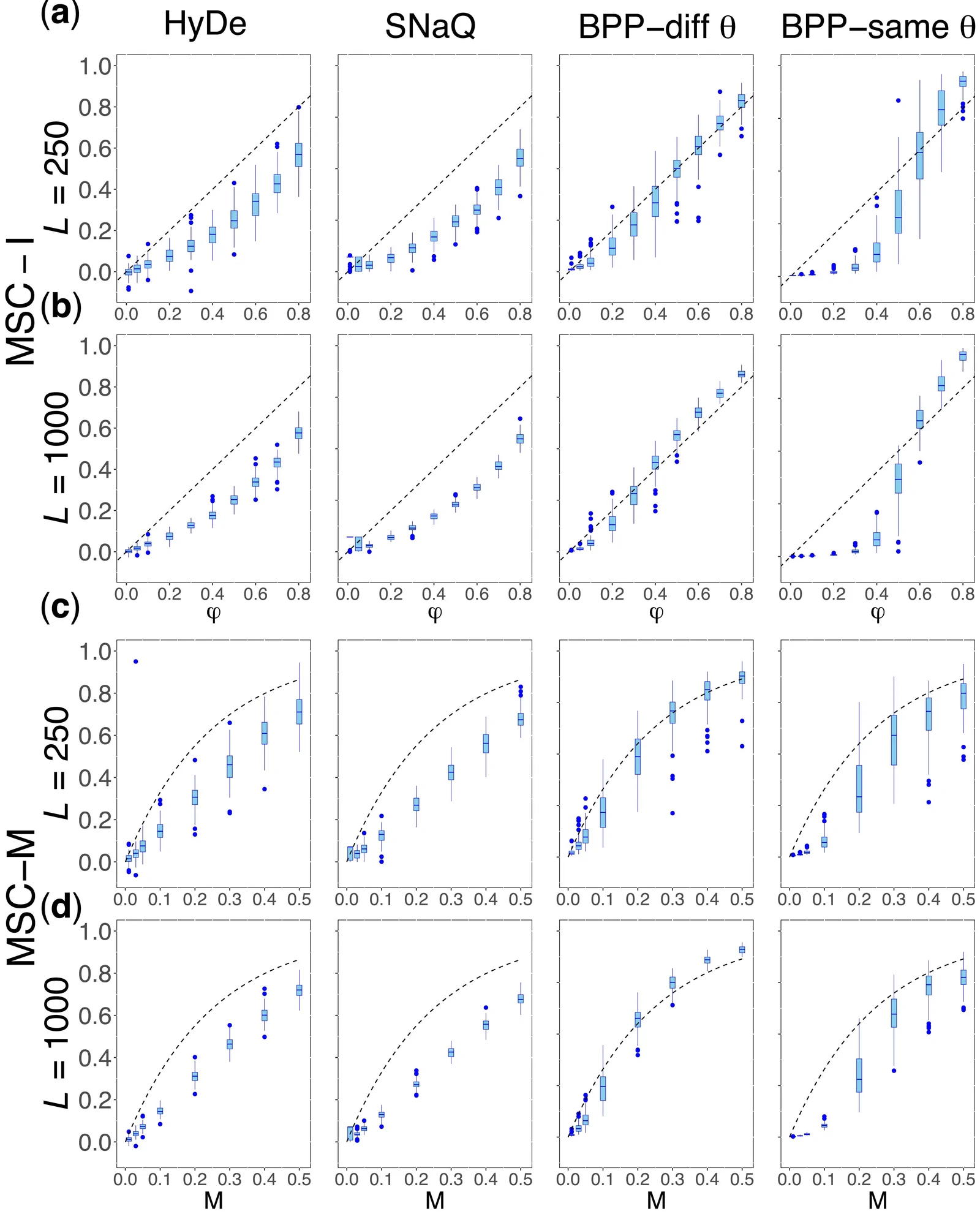
Boxplots of estimates of *φ* obtained in Hyde, SNaQ, and bpp analyses of the data of Fig. 8. See legend to Fig. 7.

In summary, incomplete taxon sampling may cause multiple branches on the phylogeny to be merged, end-to-end, into one branch that is the recipient population for gene flow, which requires a redefinition of the recipient population and its size, but this has minimal effects on Bayesian estimation of the introgression probability (i.e., accurate estimation of *φ* and poor estimation of $\theta _B$ in Fig. 8). However, incorrect assumption of one population size for all species can lead to large biases in Bayesian estimation of the rate of gene flow (Supplementary Fig. S3). Summary quartet methods assume one population size for all species and similarly produce seriously biased estimates of the rate of gene flow when population size differs among species (Fig. 9).

### Six Species: Inference of Gene Flow using *f*-Branch and bpp

The *f*-branch approach is an exploratory method for integrating triplet tests of introgression when data from many species are analyzed using summary triplet methods (Malinsky et al. 2018, 2021). The method may be affected by incomplete taxon sampling because triplet methods ignore other species which may be donors of gene flow to species in the triplet. To assess the impact of incomplete taxon sampling on inference by the *f*-branch method, we simulated sequence data under the MSC-I model for six species of Fig. 1a and analyzed the data using the *f*-branch method, implemented in Dsuite (Malinsky et al. 2021). We examine the performance of the test to infer gene-flow events as well as estimation of the introgression probabilities by the method. Two sets of data were simulated assuming either one *θ* for all populations or different *θ*s for the thin and thick branches on the species tree (Fig. 1a). The correct binary species tree (Fig. 1a) was assumed, and species *O* was used as the outgroup in all triplet analyses. For comparison, we analyze the same data using bpp, under the correct model of Fig. 1a.

#### Overview of the *f*-branch approach to inferring gene flow

Here we provide an overview of the *f*-branch procedure, which consists of the following steps.

First, a triplet method (e.g., $f_4$, Patterson et al. 2012 or HyDe, Blischak et al. 2018) is used to estimate introgression probabilities for all triplets.

Second, an attempt is made to move inferred introgression events onto ancestral branches on the species tree. Consider the species tree of Fig. 10, in which *A* and *B* are clades, and *b* is an ancestral branch. Selecting a species $A_i$ from clade *A* and another species $B_j$ from clade *B* leads to triplets of the form $A_i B_j C$, generating many *φ* estimates. The method moves the introgression event to the ancestral branch *b* with the estimate of the introgression probability given by


(15) \begin{eqnarray*}
f_b(C) \equiv \hat{\varphi }_{C\rightarrow b} = \mathrm{med}_i [\mathrm{min}_j [f(A_i, B_j; C, O)]] ,
\end{eqnarray*}

where $f(A_i, B_j; C, O)$ is the estimate of $\varphi _{C\rightarrow B}$ from the $A_i B_j C$ triplet (using, e.g., $f_4$), and where med stands for median and min for minimum (Malinsky et al. 2018).

**Figure 10.**
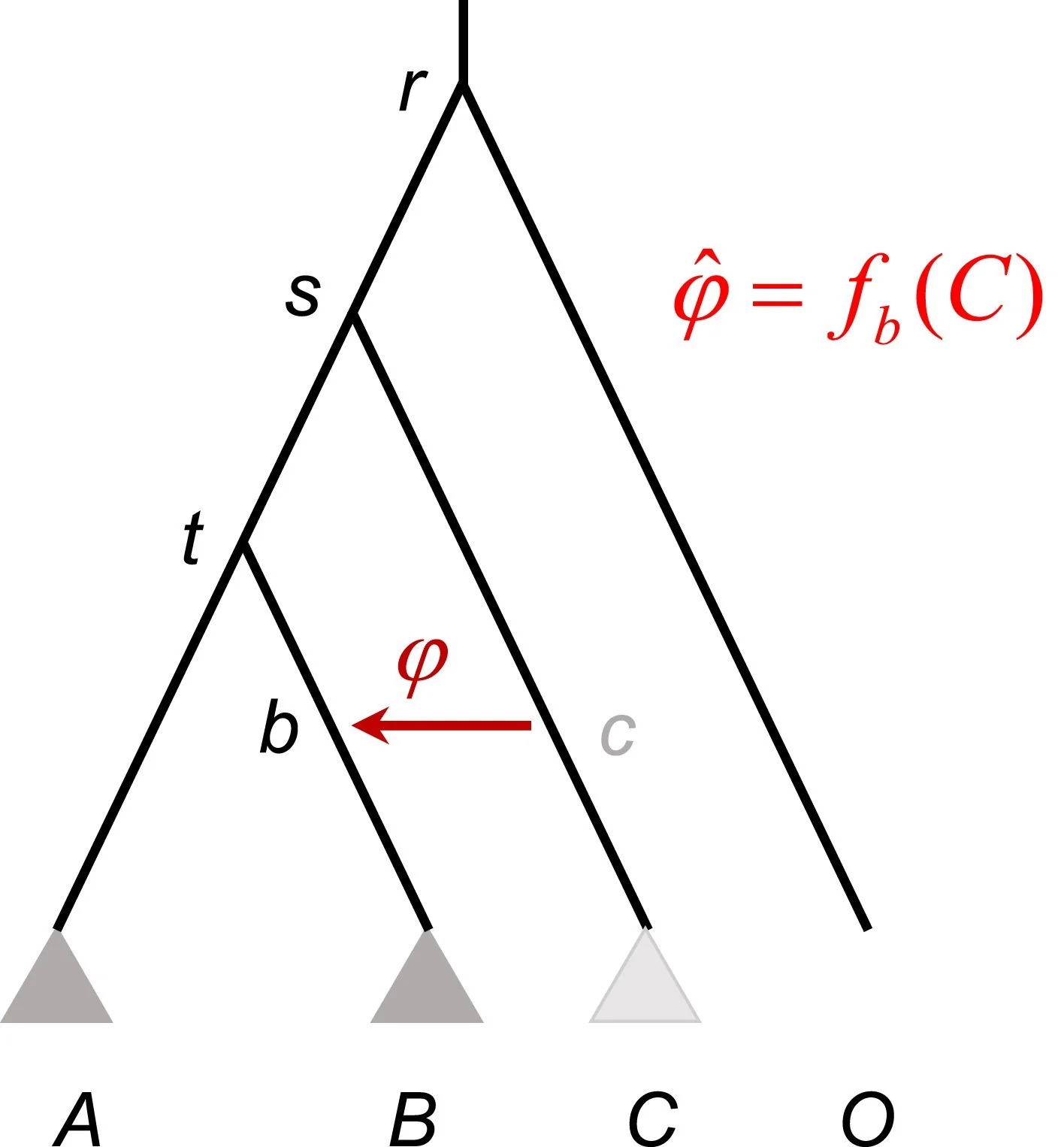
Species tree with an outgroup (*O*) to illustrate the *f*-branch approach. Selecting a species $A_i$ from clade *A* and another species $B_j$ from clade *B* leads to many triplets of the form $A_i B_j C$, with each triplet generating an *φ* estimate. The approach then integrates the triplet estimates to produce an introgression probability into the ancestral branch *b*: $f_b(C) \equiv \hat{\varphi }_{C\rightarrow b}$.

Third, estimates generated from Equation (15) are filtered via a statistical test or an empirical cut-off, and estimates that do not pass the test are reset to 0. Dsuite uses a block-jackknife procedure to estimate the standard error of the *D*-statistic, and calculates a *Z* score or *p*-value, and uses the Holm-Bonferroni correction to control the family-wise error rate (FWER) (Malinsky et al. 2021). Alternatively an empirical cut-off on *φ* may be applied, for example, 0.03 (Malinsky et al. 2018) or 0.05 (Malinsky et al. 2021). If the triplet estimate does not pass the test (e.g., if the *p*-value is *>0.01*), the *f*-branch estimate is set to 0. The rationale is that one should not claim gene flow unless the evidence is strong.

We present the *f*-branch analysis of a replicate dataset in detail in Fig. 1c, to illustrate the procedure. The analysis produced three nonzero estimates. They were, in decreasing order, $\hat{\varphi }_{F\rightarrow D} = f_D(F) = 0.212$ based on the *CDF* triplet; $\hat{\varphi }_{E\rightarrow D} = f_D(E) = 0.194$ based on *CDE*; and $\hat{\varphi }_{D\rightarrow E} = f_E(D) = 0.0194$ based on *FED* (Fig. 1c). Among the three inferred events, the *E→ D* gene flow clearly reflected the rates $\varphi _{z\rightarrow w} = 0.1$ and $\varphi _{v\rightarrow u} = 0.2$ in the true model (Fig. 1a). However the estimate (0.194) was lower than the sum $\varphi _{z\rightarrow w} + \varphi _{v\rightarrow u} = 0.3$, if one expects gene flow occurring in the same direction to be accumulative (see, e.g., Malinsky et al. 2021, Fig. 1c). The inferred *D→ E* gene flow did not exist in the true model and reflected the fact that the triplet methods assume an inflow setup (e.g., Ji et al. 2023) and are unable to infer the direction of gene flow. Finally the inferred *F→ D* gene-flow event reflected the true *v→ u* gene flow, and to a lesser extent the *z→ w* event as well since species *E* is an unsampled ghost species in the analysis of the *CDF* triplet. It is interesting that $\hat{\varphi }_{F\rightarrow D} > \hat{\varphi }_{E\rightarrow D}$.

The *x→ y* introgression in the true model (Fig. 1a) was not inferred in the *f*-branch analysis of the dataset. Note that given the true model, there exists no triplet in which the *x→ y* gene flow is ‘inflow’, as assumed by the triplet methods. While the *x→ y* event does not fit the inflow setup, gene-flow signal in the opposite direction (*y→ x*) was detected in the following four triplets: *ECA*, *FCA*, *EDA*, and *FDA*, with the $f_4$ estimates to be 0.0015, 0.0017, 0.0032, and 0.0034, respectively. However, those estimates were not significant (with the *p*-value *>0.01*) and were set to zero. Thus no gene flow, either from *x→ y* or from *y→ x*, was inferred in the *f*-branch analysis of the dataset (Fig. 1c).

#### Performance of the *f*-branch test

The performance of a statistical test is assessed by its false-positive rate (type-I error) and power (or 1 minus the false-negative rate). A useful test is required to have the type-I error under control (with the false positive rate to be $\le 5\%$ if the test is conducted at the 5% level), and then one evaluates the power of the test. If we insist on the inference of the full model of Fig. 1a, including the correct number of gene-flow events, correct identification of populations involved in gene flow, and correct directions of gene flow, the approach achieved 0% accuracy: in none of the replicate datasets simulated in this study was the correct model ever recovered by the method.

However, there is ambiguity in deciding whether a gene-flow event inferred by the *f*-branch approach is correct or not. For example, as the triplet methods used are agnostic of the direction of gene flow, an inferred gene flow event may arguably be considered correct if it involves the correct lineages but wrong direction. Indeed Malinsky et al. (2021, p.592) point out that “an *f*-branch result in itself does not indicate directionality of gene flow”, although estimates of introgression probabilities for unidirectional gene flow are reported (e.g., Fig. 3 of the same paper). The probability of introgression is meaningful only if the direction of gene flow is known. Furthermore all triplet methods used in *f*-branch to estimate the introgression probability assume inflow. Here we consider the *f*-branch estimates as meaningful, noting that the estimates may be biased if gene flow is in the opposite (outflow) direction.

If the inferred *A→ C* and *B→ C* gene flow is considered a false positive error, the error rate was no larger than 5% at the 0.03 cut-off, and even lower at the 0.05 cut-off (Table 2). If we consider those gene-flow events as correct results (reflecting the true *x→ y* introgression in the opposite direction, Fig. 1a), those proportions will be the power of the test, suggesting that power was very low.

**Table 2.** False positive rates and power of *f*-branch test of gene flow in analysis of data simulated under the model of Fig. 1

	Same *θ*		Different *θ*	
Cutoff $^\dagger$	0.03	0.05	0.03	0.05
**False positive rates**				
$f_C(A)$	0%	0%	0%	0%
$f_C(B)$	0%	0%	1%	0%
Other branches	$\le 1\%$	0%	$\le 1\%$	0%
**Power**				
$f_U(A)$ (*x→ y*)	45%	0%	0%	0%
$f_U(B)$ (*x→ y*)	44%	0%	0%	0%
$\mathrm{med}(f_U(A),f_U(B))$ (*x→ y*)	42%	0%	0%	0%
$f_E(D)$ (*z→ w*)	6%	0%	0%	0%
$f_D(E)$ ($v\rightarrow u, z\rightarrow w$)	100%	100%	100%	100%
$f_D(F)$ (*v→ u*)	100%	100%	100%	100%
$\mathrm{med}(f_D(E),f_D(F))$ (*u → v*)	100%	100%	100%	100%

$^\dagger$ Evidence for introgression is considered significant if the estimated introgression probability exceeds an empirical cut-off, i.e., if $f_b > 0.03$(Malinsky et al. 2018) or $f_b > 0.05$ (Malinsky et al. 2021).

The method detected the *A→ U* and *B→ U* gene-flow events at the 0.03 cut-off in $\sim 45\%$ of datasets simulated with the same *θ* for all populations, and in 0% of datasets simulated with different *θ*s (Table 2). These gene-flow events reflected the true gene flow from *x→ y* in the opposite direction involving the common ancestor of *A* and *B* (Fig. 1a). Note that the *f*-branch approach detects gene flow from donor populations that are tips but not internal nodes on the species tree. If we consider those events as correct results, both reflecting the *x→ y* gene flow, the power for detecting the event is then 45% and 0%. If we consider the inferred *A→ U* and *B→ U* gene-flow events to be incorrect, those proportions will be false positive rates (Table 2).

The assumption that the donor population for gene flow must be a tip branch may be problematic. One idea may be to move the gene-flow events supported by all descendant tips onto the ancestral branch, similar to the *f*-branch treatment of recipient populations. Suppose one chooses species $A_i, B_j, C_k$ from clades *A,B,C* to form the triplet $A_i B_j C_k$, with estimated introgression probability $f(A_i, B_j; C_k, O)$ (Fig. 10). One may generate an estimate for the *c→ b* gene flow involving the ancestral branches *c* and *b*,


(16) \begin{eqnarray*}
\hat{\varphi }_{c\rightarrow b} \equiv f_b(c) = \mathrm{med}_k\mathrm{med}_i \mathrm{min}_j f(A_i, B_j; C_k, O) ,
\end{eqnarray*}

where the $\mathrm{med}_k$ operation is taken only if all rates involving descendent tips of branch *c* are positive. Note that in the dataset of Fig. 1c, this approach will infer the *v→ u* gene flow but miss the *z→ w* gene flow.

If we apply this idea and replace the *A→ U* and *B→ U* gene-flow events by *y→ x* gene flow, we will infer the correct lineages (the true event is from *x→ y* in the opposite direction). Then power at the 0.03 cut-off will be 42% in datasets assuming the same *θ* and 0% in datasets with different *θ*s (Table 2).

The *E→ D* and *F→ D* gene-flow events were detected in all datasets (Table 2). As noted in Fig. 1c, the *F→ D* event reflected the true *v→ u* event, and to a lesser extent the *z→ w* event as well since species *E* is a ghost species in the analysis of the *CDF* triplet. The power for detecting both *E→ D* and *F→ D* gene-flow events was 100%.

The *D→ E* event was detected at the 0.03 cut-off in 6% of datasets simulated with the same *θ* and in 0% of datasets with different *θ*s. This reflected the true *E→ D* gene flow (Fig. 1a) and may be considered a correct result.

The *v→ u* introgression in the true model involves the common ancestor of *E* and *F* as the donor population. This was not detected, because as discussed above the *f*-branch approach assumes that the donor population must be a tip branch on the species tree.

#### Performance of *f*-branch estimation of introgression probabilities

Estimates of introgression probabilities obtained from *f*-branch analysis of replicate datasets simulated under the model of Fig. 1a are summarized in Fig. 11. Five gene-flow events were ever detected by the *f*-branch method (Fig. 11): $f_{U}(A)$ (or $\varphi _{A\rightarrow U}$), $f_{U}(B)$ (or $\varphi _{B\rightarrow U}$), $f_D(E)$ (for $\varphi _{E\rightarrow D}$), $f_D(F)$ (for $\varphi _{F\rightarrow D}$), and $f_E(D)$ (for $\varphi _{D\rightarrow E}$). The first two events, *A→ U* and *B→ U*, reflect the *x→ y* gene flow in the true model although the true direction is the opposite and the true lineage is a parental lineage (Fig. 1a). In general the estimated rate for those two events were low, except for datasets simulated under the same *θ* (Fig. 11). The assumed wrong direction of gene flow may partly account for the low estimates, and the truncation used by Dsuite, which sets the $f_4$ estimate of *φ* for any triplet to zero if the associated *D*-statistic is not significant (Malinsky et al. 2021), may be another factor.

**Figure 11.**
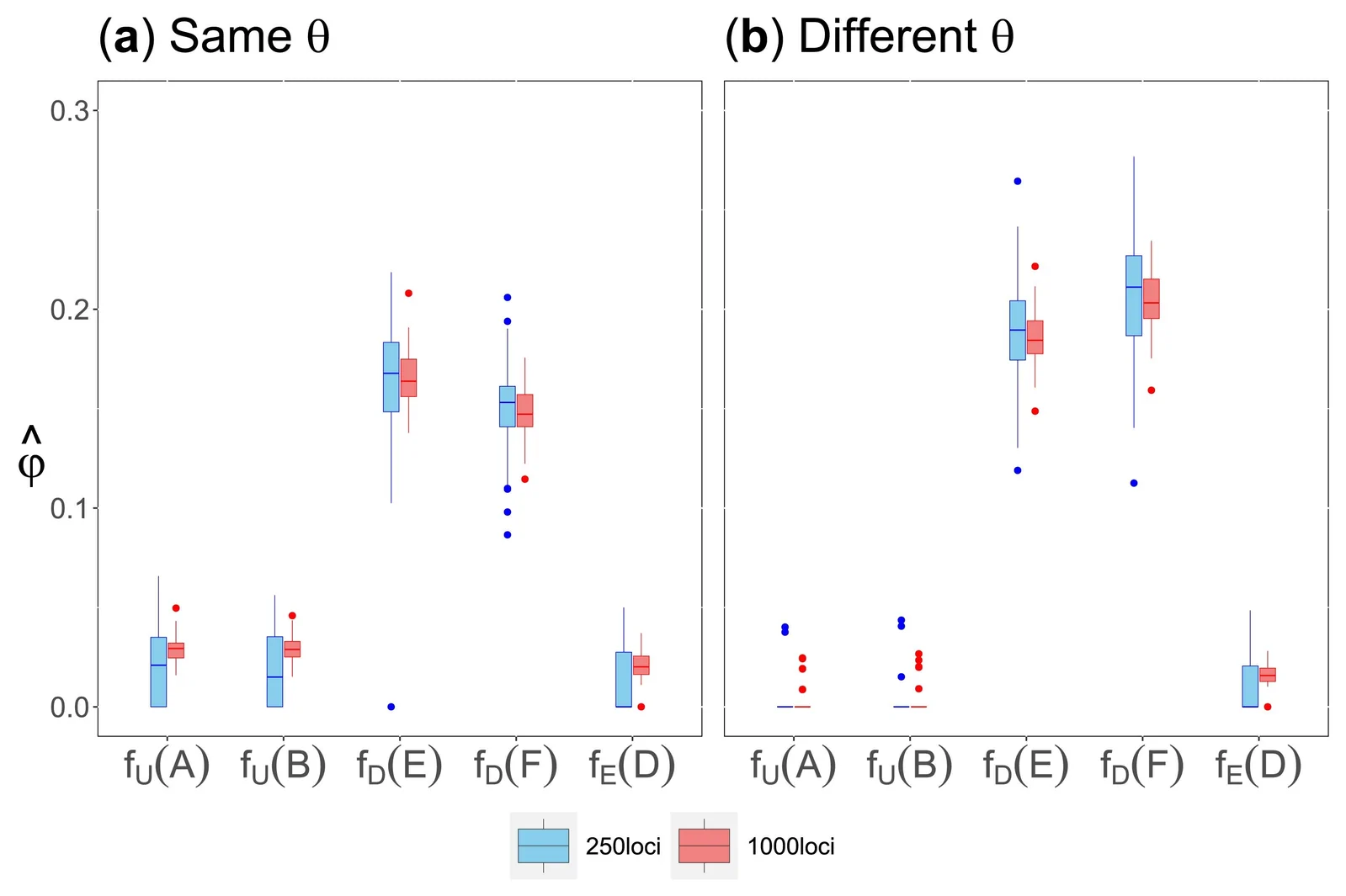
Boxplots of estimates of introgression probability (*φ*) from the *f*-branch analyses of data simulated under the six-species model of Fig. 1a, with either *L=250* or 1000 loci in each dataset. Only five introgression events were ever found to be significant (with FWER *< 0.001*) in the data simulated in this study.

The other three events, *E→ D*, *F→ D*, and *D→ E*, all reflect the true gene flow from *E→ D* (that is, *z→ w* and also *v→ u*; Fig. 1a).

We consider the *F→ D* introgression to be a correct result as well; the detected signal may reflect both the *V→ D* and *E→ D* gene flow involving the sister species *E* of *F*. If the introgression probabilities are additive and independent of the direction, one would expect $\varphi _{E\rightarrow D}$ to be close to $\varphi _v + \varphi _w = 0.2+0.1=0.3$. Estimates from *f*-branch were around 0.1, much lower. The $f_D(F)$ estimates were smaller than and close to 0.1, also much lower than $\varphi _v = 0.2$.

#### Analysis of the six-species data using bpp

We analyzed the data simulated under the six-species model with three introgression events (Fig. 1a) using bpp, specifying the correct MSC-I model. The posterior means and the 95% HPD CIs for parameters in the true model were well behaved (Supplementary Fig. S4). The CIs were narrower in large datasets of *L=1000* loci than in small datasets (*L=250*). The estimates were far more precise with much narrow CIs in datasets simulated under the same *θ* for all populations than for datasets with different *θ*s: if all species on the phylogeny have the same population size (*θ*) there is a large benefit in parameter estimation in enforcing the assumption.

We calculated the Bayes factor in support of gene flow ($B_{10}$) using the S-D density ratio (Ji et al. 2023). In every dataset, the Bayes factor for testing each of the three introgression events was *∞* (except for one dataset of 250 loci), strongly supporting gene flow.

We also analyzed the *CDE* triplet data (including the outgroup *O*). We used the data simulated under the six-species model with *L = 1000* loci, to fit four variants of the MSC-I model: (i) the true model with two introgression events (*v→ u*, *z→ w*; Fig. 1b), (ii) bidirectional introgression (BDI, *z ↔ w*), (iii) unidirectional introgression (UDI) from *E→ D* (or *z→ w*), and (iv) UDI in the wrong direction from *D→ E* (or *w→ z*). Estimates of the introgression probabilities are summarized in Table 3.

**Table 3.** Average posterior means and 95% HPD CIs for introgression probabilities (*φ*) in bpp analyses of the *CDE* triplet data (including outgroup *O*) simulated under the six-species model of Fig. 1a

Introgression model	$\hat{\varphi }_{v\rightarrow u}$	$\hat{\varphi }_{z\rightarrow w}$
**One *θ* for all populations**		
(i) *v → u* & *z → w*	0.195 (0.130, 0.258)	0.111 (0.055, 0.168)
(ii) *z ↔ w*	NA	0.243 (0.210, 0.276)
	NA	0.004 (0.000, 0.011)
(iii) *z → w*	NA	0.244 (0.211, 0.277)
(iv) *w → z*	0.388 (0.331, 0.446)	NA
**Different *θ***s		
(i) *v → u* & *z → w*	0.194 (0.136, 0.250)	0.108 (0.054, 0.162)
(ii) *z ↔ w*	NA	0.249 (0.218, 0.281)
	NA	0.004 (0.000, 0.012)
(iii) *z → w*	NA	0.250 (0.219, 0.282)
(iv) *w → z*	0.524 (0.445, 0.606)	NA

Note.— Data are simulated and analyzed assuming either one *θ* or different *θ*s for populations. The number of loci is *L = 1000*. Each dataset is analyzed under four MSC-I models, each with one or two introgression events. Model i is the true model of Fig. 1b, with two unidirectional introgression (UDI) events (*v→ u* and *z→ w*), model ii assumes bidirectional introgression (BDI), with *z→ w* and *w→ z* introgressions occurring at the same time, model iii assumes UDI with *z→ w* introgression, and model iv assumes UDI in the opposite (wrong) direction (*w→ z*).

Model i, with two UDI gene-flow events (*u→ v* and *z→ w*), constitutes the true model for those data. Under this model the estimates were highly accurate. For example the average posterior means for $\varphi _{v\rightarrow u}$ (with true value 0.2) were 0.195 for datasets with the same *θ* and 0.194 for datasets with different *θ*s, while the corresponding estimates for $\varphi _{z\rightarrow w}$ (with true value 0.1) were 0.111 and 0.108, respectively. The estimates were slightly less precise than those from the full data for all six species (Supplementary Fig. S4) due to reduced information content. Model ii (BDI) assumes introgression in both directions (*z↔ w*): the estimates were $\hat{\varphi }_{z\rightarrow w} = 0.243$ or 0.249 in the correct direction and $\hat{\varphi }_{w\rightarrow z} = 0.004$ in the wrong direction. The estimates were largely the same as those from model iii assuming the *z→ w* introgression only. This estimated rate was somewhat accumulative (compare 0.243 with the true rate $\varphi _{v\rightarrow u} + \varphi _{w\rightarrow z} = 0.2+0.1 = 0.3$. Model iv assumes one introgression event in the wrong direction, and the estimated rate was even higher than the true rate in the correct direction ($\hat{\varphi }_{z\rightarrow w} = 0.388$ for data of the same *θ* and 0.524 for data of different *θ*s, compared with $\varphi _{v\rightarrow u} + \varphi _{w\rightarrow z} = 0.3$).

Bayes factors strongly supported gene flow in each of the four models of Table 3 except the *w→ z* gene flow in model ii (BDI), in which the gene-flow event was strongly rejected (with $B_{10}<0.01$). Note that in model iv with the only assumed gene flow in the wrong direction, gene flow was also strongly supported, consistent with the large estimate of the rate.

Those results are consistent with Thawornwattana et al. (2023). In particular, if introgression is assumed to occur in the wrong direction, the test of gene flow will often be significant and the estimated rate may even be greater than the true rate in the opposite direction. However, if bidirectional introgression is assumed, gene flow in the correct direction will be detected and the nonexistent gene flow in the wrong direction will be rejected.

#### Summary on the *f*-branch approach to inferring gene flow

Our evaluation here suggests that if there are multiple introgression events involving ancestral branches on the phylogeny, the *f*-branch test will be unlikely to infer the correct introgression model. The approach is limited by the shortcomings of the summary triplet methods used, such as the *D*-statistic, $f_4$ (Green et al. 2010, SOM18), $f_d$ ( Martin et al. 2015, Fig. 1) and HyDe ( Blischak et al. 2018, Fig. 1). Those methods are unable to identify gene flow between sister lineages, and produce biased estimates of the introgression probability if gene flow is in the opposite (outflow) direction or if population size differs among species (Fig. 9). Also the *f*-branch approach does not detect gene flow from an ancestral donor branch. We suspect that the *f*-branch test will have improved performance at inferring gene flow if more powerful and reliable triplet methods are used instead.

The *f*-branch estimates of the introgression probability, based on estimates from triplet methods ($f_4$, $f_d$ and HyDe), all of which assume inflow, may be biased if gene flow is in the opposite (outflow) direction or if it is in both directions. Note that introgressions in opposite directions between the same two lineages may neither cancel out nor be additive (Thawornwattana et al. 2023). Setting triplet estimates to zero when the test is not significant also leads to underestimation. We suggest that if a model of introgression is constructed using *f*-branch, a reliable method such as bpp should be applied to re-estimate the introgression probabilities.

## Conclusions

In this paper, we have evaluated two effects of incomplete taxon sampling: (i) inference of introgression from unsampled ghost lineages or selection of models of gene flow and (ii) estimation of the rate (or magnitude) of gene flow influenced by unsampled lineages. We note that our test of models of gene flow including ghost introgression has very limited scope, following the design of Pang and Zhang (2024) (Fig. 2). The case of ghost introgression is highly complex, concerning the size and shape of the species phylogeny, the number of gene-flow events, the lineages involved, the direction of gene flow, the existence and number of ghost lineages and their placements on the species phylogeny, etc. We leave it to the future to explore different scenarios of ghost introgression, and to develop MCMC algorithms for searching in the space of models of gene flow.

Below we summarize the major findings from our study.

We have described a Bayesian testing approach to inferring gene flow on a species triplet. Our simulation suggests that the approach can distinguish different models of gene flow such as ghost introgression, inflow, and outflow, as well as the model of no gene flow (Fig. 2), with excellent sensitivity (high power) and specificity (low false positives) (table 1). We note that the general model of Fig. 2e includes other models as special cases, including those with more than one introgression event, although our simulation assumed either no gene flow or only one event (2a–d). Good performance may be expected in datasets simulated under any model that is a special case of the general model. It may be worth investigating whether the bpp analysis of triplets can be used to replace summary triplet methods used in the *f*-branch approach.
Summary triplet methods do not have the power to distinguish different models of gene flow (such as ghost introgression, inflow, and outflow), and have high false-positive rates (Table 1 and Pang and Zhang 2024). Incomplete taxon sampling may cause gene flow between non-sister species to become gene flow between sister species, which is unidentifiable using summary triplet methods.
When the population size for a species changes over time (as in Fig. 3a) but is assumed to be constant, the estimated population size from genomic data reflects the weighted average, with the weight to be the number or proportion of coalescent events (Equations 5 and 8).
When the population size for the recipient species changes over time or when incomplete taxon sampling causes a redefinition of the recipient population, the Bayesian method can still provide reliable estimation of the introgression probability (Figs. 6 and reffig:4s-diff-diff-bpp). In other words, genomic data allows reliable estimation of the proportion of immigrants in the recipient population ($\varphi _{xy}$) even though the number of immigrants ($N_y\varphi _{xy}$) and the population size for the recipient lineage ($N_y$) may be poorly estimated. This is because genealogical histories are influenced by the proportion of immigrants in the recipient population, not by the number of immigrants.
Assuming the same population size for all species when population size differs among species may cause both the Bayesian method and summary triplet methods to produce seriously biased estimates of the rate of gene flow (Supplementary Fig. S3 and Fig. 9). Thus for inference of gene flow in the MSC framework, it appears to be important to account for population size differences between species but not so important to account for temporal changes in population size for the same species (within one branch on the species tree).
The *f*-branch method is effective at generating plausible gene-flow scenarios when there are overwhelmingly many triplets with significant gene flow. However, the inferred gene-flow model is highly likely to be incorrect if there are multiple introgression events involving ancestral lineages on the phylogeny. The *f*-branch approach is limited by the shortcomings of the summary triplet methods, such as inability to infer gene flow between sister lineages and biased estimation of introgression probabilities if gene flow is not in the inflow direction or if population size differs among species (Fig. 9). Also the *f*-branch approach cannot infer gene flow from an ancestral donor branch. Use of powerful and reliable triplet methods is likely to lead to improved performance of the *f*-branch test.
The *f*-branch method tends to underestimate introgression probabilities when gene-flow events are correctly identified. If a model of introgression is constructed using *f*-branch, it is advisable to use a more reliable method such as bpp to re-estimate the introgression probabilities.

## Supplementary Material

syag023_Supplemental_File

## Data Availability

Data available from the Dryad Digital Repository: https://doi.org/10.5061/dryad.cvdncjtgt.
