# Supplementary material for: The Impact of Incomplete Taxon Sampling on Inference of Gene Flow by Bayesian and Summary Methods Using Genomic Sequence Data
Source: Syst Biol. 2026 Mar 3;75(6):1097–119. doi: 10.1093/sysbio/syag023 (PMC13637633; doi:10.1093/sysbio/syag023)
Supplement: Supplementary file 1 [file syag023_supplemental_file.pdf]

Supplemental Information

The impact of incomplete taxon sampling on inference of gene flow by Bayesian and summary methods using genomic sequence data

Sirui Cheng, Tomáš Flouri, Tianqi Zhu and Ziheng Yang

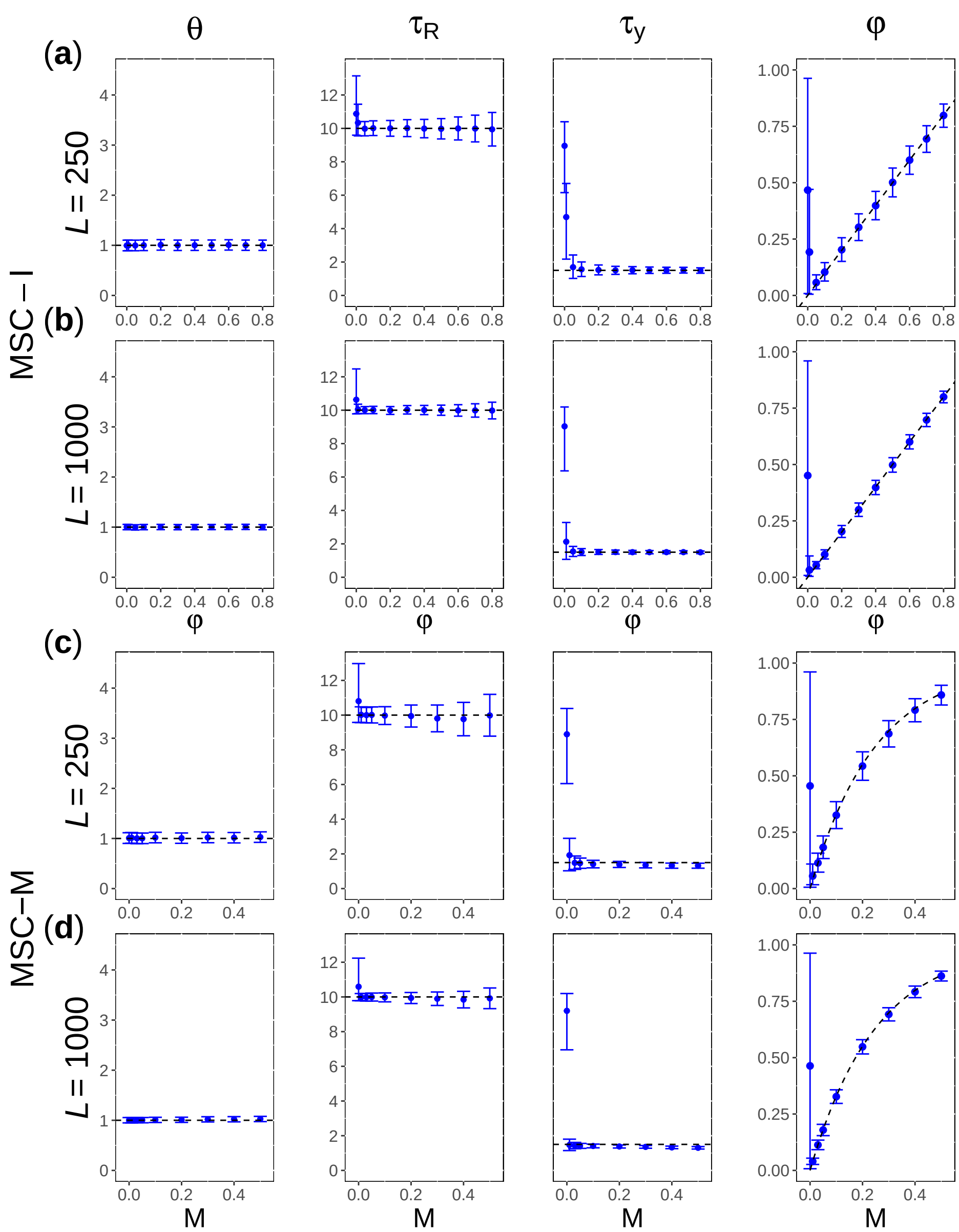

Fig. S1: [2s-same-same] (**a**, **b**) Average posterior means and the 95% HPD CIs of parameters when the data are simulated and analyzed under the MSC-I model (Fig. 4**a**) assuming the same θ for all species, plotted against the introgression probability (φ). (**c**, **d**) Parameter estimates under MSC-I when data are simulated under MSC-M (Fig. 4**b**), plotted against the migration rate (M). Estimates of θ and τ are mutipled by 1000. Black dashed lines represent the true value in **a&b** or expected value (φ_0) in **c&d**.

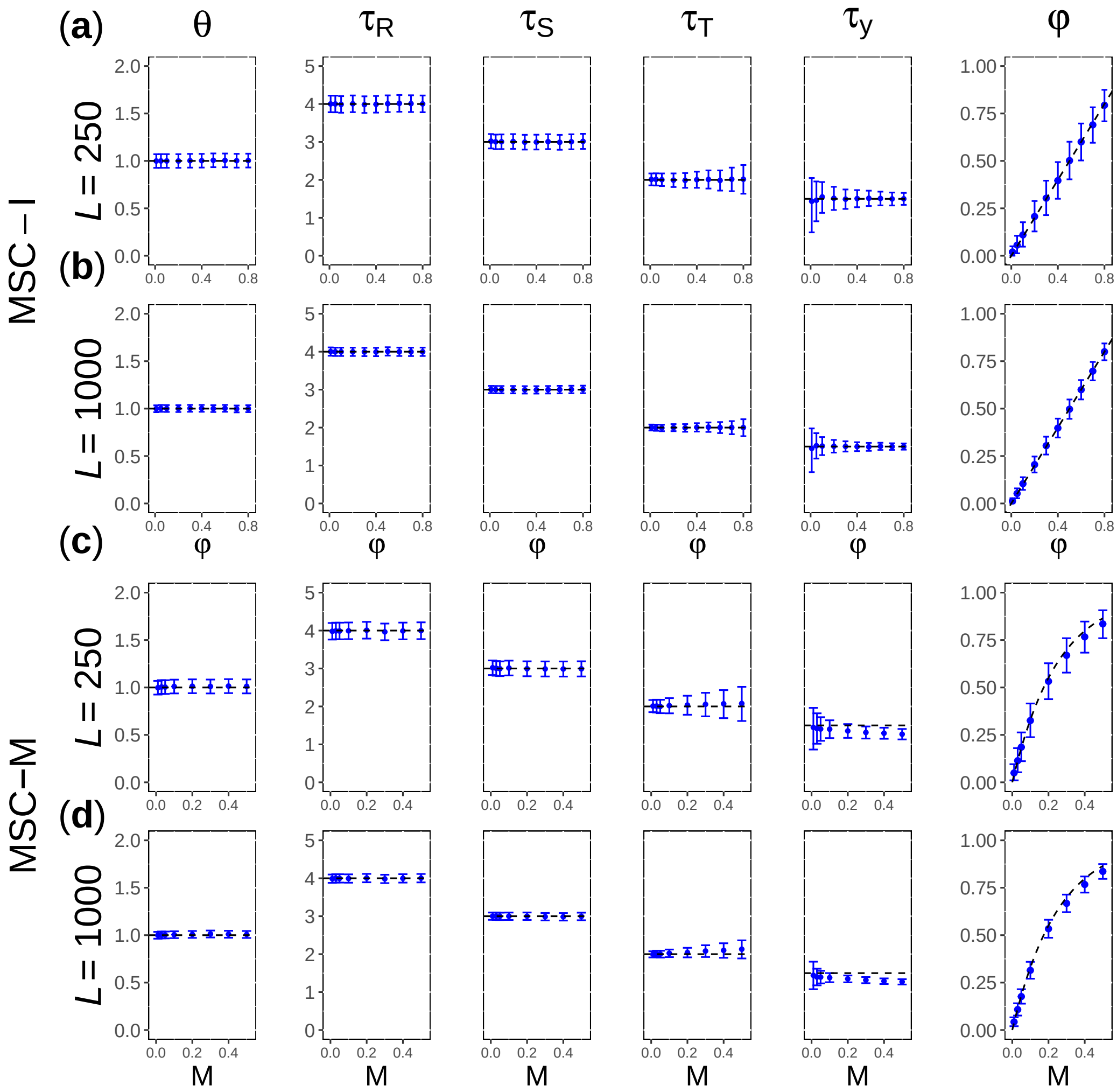

Fig. S2: [4s-same-same] (**a**, **b**) Average posterior means and the 95% HPD CIs of parameters from data simulated and analyzed under the MSC-I model (Fig. 5**a**) assuming the same θ for all populations, plotted against the introgression probability (φ). (**c**, **d**) Parameter estimates under MSC-I from data simulated under MSC-M (Fig. 5**b**), plotted against the migration rate (M). Dashed lines represent true parameter values except that in **c&d** for τ_y and φ, they represent $(\tau_T + \tau_U)/2$ and φ_0 (eq. 14), respectively.

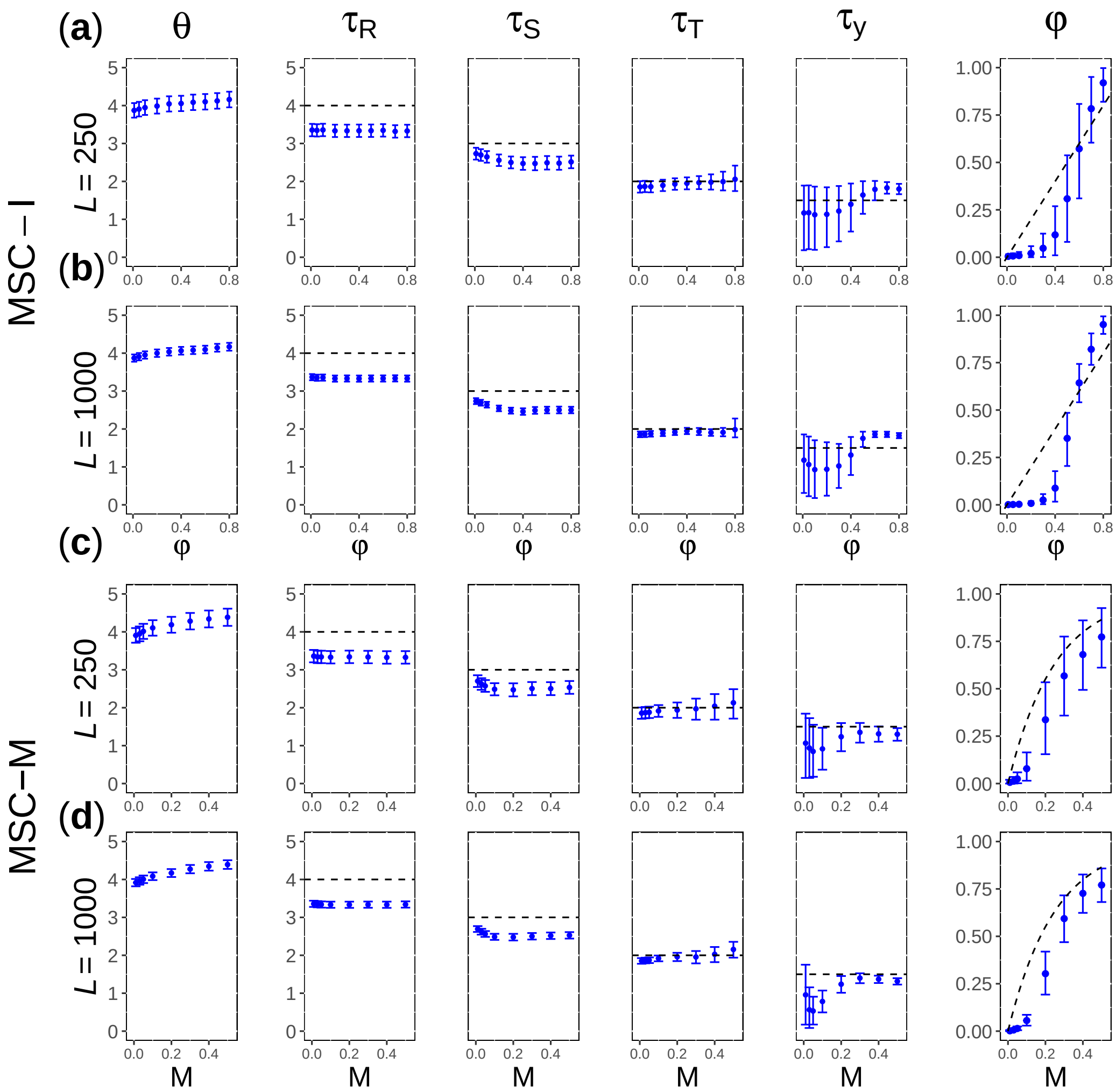

Fig. S3: [4s-diff-same] (**a**, **b**) Average posterior means and the 95% HPD CIs of parameters under MSC-I (Fig. 5**a**) from data simulated under the MSC-I model (Fig. 5**a**) with different θs for species, but assuming the same θ for all populations in data analysis. (**c**, **d**) Estimates under MSC-I when the data are simulated under the MSC-M model (Fig. 5**b**)

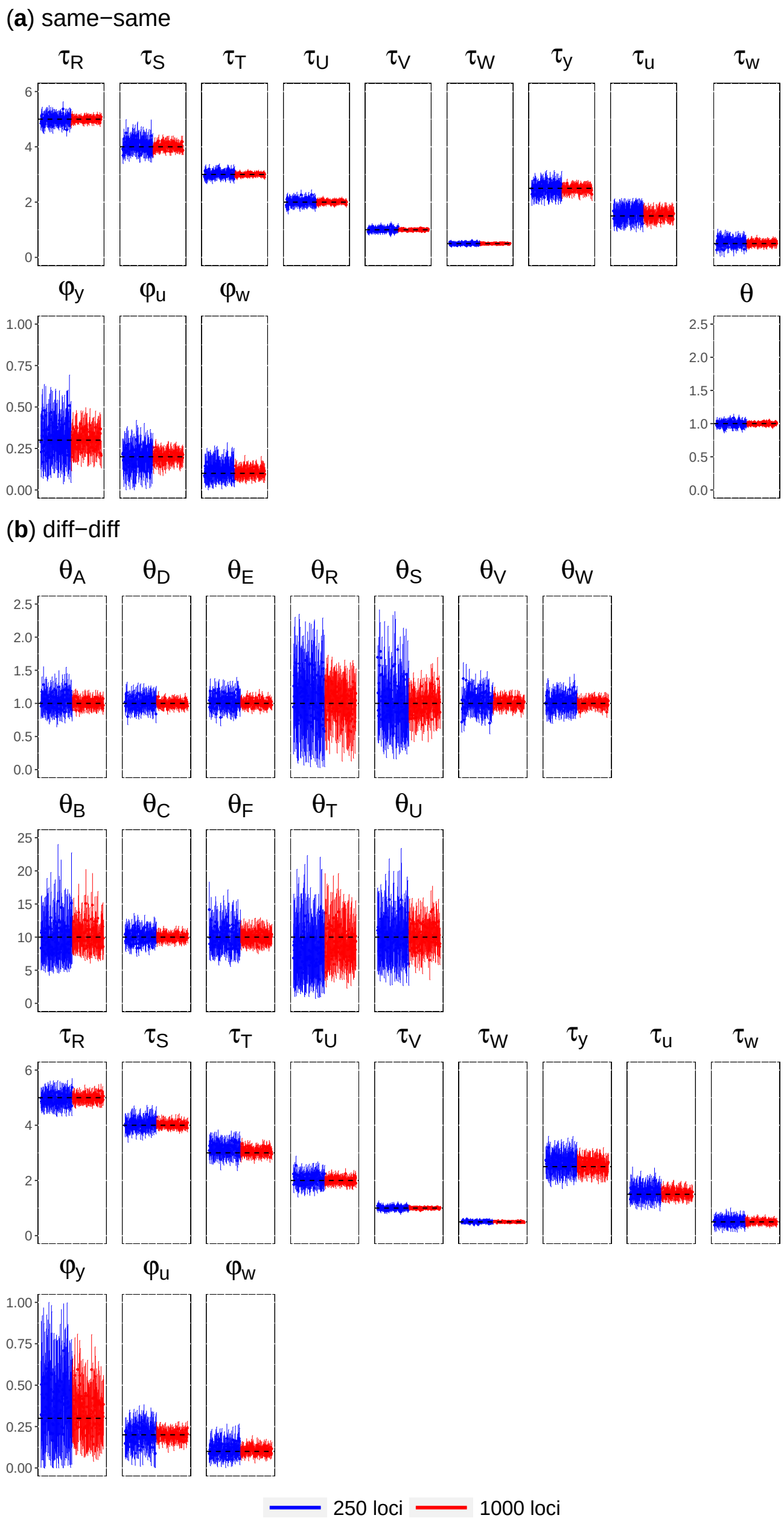

Fig. S4: Posterior means and the 95% HPD CIs for parameters when replicate datasets are simulated and analyzed under the MSC-I model of Figure 1**a** assuming either (**a**) the same θ for all populations or (**b**) different θ for populations. Black dashed lines represent the true values.
